# *Ziziphus* Seed-Based Adsorbents: A Review Study on Preparation, Adsorption Mechanisms, and Environmental Applications

**DOI:** 10.3390/molecules31193556

**Published:** 2026-10-06

**Authors:** Zinab Alawa, Hazim Qiblawey

**Affiliations:** Department of Chemical Engineering, Qatar University, Doha P.O. Box 2713, Qatar; za1701664@student.qu.edu.qa

**Keywords:** *Ziziphus* spp., seed-derived adsorbent, activated carbon, adsorption, wastewater treatment, adsorption mechanism, characterization, environmental remediation

## Abstract

*Ziziphus* seeds, an abundant and low-cost lignocellulosic residue generated across the value chains of *Z. mauritiana*, *Z. spina-christi*, and *Z. jujuba* in arid and semi-arid regions, have emerged as a promising but under-synthesized feedstock for adsorption-based water treatment. This review provides a critical, mechanism-focused assessment of *Ziziphus* seed-derived adsorbents—raw biomass, biochars, activated carbons, and magnetic or nanocomposite hybrids—covering preparation routes, physicochemical characterization, adsorption mechanisms, and environmental performance for dye and heavy metal remediation. Comparative analysis of the literature (2020–2025) shows that adsorption capacity is dictated primarily by the intensity and nature of thermochemical/chemical modification rather than by botanical origin alone: chemical activation with H_3_PO_4_, H_2_SO_4_, or ZnCl_2_ and alkali or magnetic functionalization yield specific surface areas ranging from approximately 100 to 1900 m^2^ g^−1^ and substantially enhance dye and metal uptake relative to unmodified biomass. Mechanistic evidence indicates that dye removal is governed predominantly by electrostatic attraction, hydrogen bonding, π–π interactions, and pore filling, whereas heavy metal sequestration (particularly Cr(VI), Cd(II), and Pb(II)) proceeds via surface complexation, ion exchange, and, in some systems, redox transformation, with markedly pollutant-specific pH optima. Kinetic and equilibrium data are best described by pseudo-second-order and Freundlich models for heterogeneous carbons and metal systems, while pseudo-first-order and Langmuir behavior are more prevalent for rapidly adsorbing dyes and structurally hybridized materials; thermodynamic parameters confirm spontaneous, but mechanistically variable (endothermic or exothermic), adsorption. Despite promising bench-scale capacities, including magnetic biochars achieving >98% Cr(VI) removal, the field remains constrained by an overreliance on idealized single-solute batch systems and negligible data on the removal of nutrients, pharmaceuticals, and per- and polyfluoroalkyl substances. Future research should prioritize application-specific functionalization, continuous-flow and multicomponent testing, long-term regeneration studies, and integrated sustainability assessment to advance *Ziziphus*-derived adsorbents toward field-scale deployment.

## 1. Introduction

Water pollution is one of the most chronic environmental problems, as industrial, agricultural and domestic activities are constantly releasing toxic pollutants into water bodies [1]. Heavy metals, dyes, pharmaceuticals, pesticides and related emerging contaminants are consistently recognized in recent reviews as major pollutant classes that pose a threat to ecosystems and human health due to their toxicity, persistence, bioaccumulation (for metals) and non-biodegradability (for dyes and pharmaceuticals) [2,3]. In particular, the effluents from the textile industry and allied industries often contain both dye molecules and toxic metal ions, leading to chemically complex effluents that are hard to treat with a single traditional treatment method [4]. Overall, the pollution load keeps increasing, while available treatment technologies are ineffective and neither affordable nor environmentally friendly [5,6]. Although conventional wastewater treatment technologies remain essential, recent literature has documented their shortcomings. While high removal efficiencies can be achieved for certain pollutants using coagulation, flocculation, precipitation, flotation, ion exchange, membrane processes, and advanced oxidation, the use of these methods is often limited by their high costs, energy consumption, complexity, sludge production, or pollutant-specific performance [7,8]. For specific contaminants, advanced oxidation processes (AOPs) can be very good, but they can also consume a significant amount of energy and are not a universal solution for mixed wastewater [9]. In spite of the good performance of commercial activated carbon, its cost and limited ability to be fully regenerated still hinder its use in broader applications, particularly in resource-limited environments [10]. In recent years, the demand for treatment systems that offer high pollutant removal efficiency at reduced material cost, low environmental impact, and high compatibility with circular resource use has been growing accordingly [11].

In this context, adsorption has been one of the most consistently preferred water treatment technologies due to its ease of operation, versatility and relatively low cost [12]. Adsorption has recently been reported as being low-cost, sustainable, user-friendly and effective in removing both inorganic and organic contaminants from aqueous media [13,14]. However, its effectiveness is highly dependent on the choice of adsorbent, since the surface area, porosity, number of sorption sites available, and the chemistry of the functional groups play critical roles. Also, the solution conditions (pH, contact time, initial concentration, temperature, and coexisting ions) will dictate the adsorption capacity and selectivity [15,16]. From a mechanistic point of view, the recent literature indicates that the uptake process of pollutants by bio-derived adsorbents can be described as a complex process that involves a combination of pore filling, electrostatic attraction, ion exchange, hydrogen bonding, π–π interactions, complexation and diffusion-controlled transport, depending on the nature of the pollutant and the adsorbent [17].

Agricultural biomass wastes have emerged as focal points in this search, as they are abundant, renewable, cheap and chemically appropriate for use as functional sorbents [18,19]. In recent years, numerous studies have pointed to the possibility of using crop residues, peels, shells, stones, husks, fibers, and other agro-wastes, directly after drying and grinding, through chemical or physical modification, or by carbonizing and activating them to produce biochar and activated carbon [20]. Their usefulness stems from their lignocellulosic structure, which contains hydroxyl, carboxyl, carbonyl, phenolic, amino and other functional groups that can bind pollutants via various interactions [21,22]. These materials also adhere to the principles of green chemistry and the circular economy, which emphasize the valorization of waste that would otherwise be discarded or incinerated or contribute to local pollution burdens [23]. Meanwhile, it has been reported that raw biomass generally has poor adsorption performance compared to engineered commercial adsorbents, prompting the activation, impregnation, magnetization and composite formation of raw biomass to enhance its porosity, surface reactivity and regeneration behavior [4,24]. *Ziziphus* seeds are emerging as a promising but under-synthesized adsorbent platform among the different agricultural precursors studied. In the recent literature, *Ziziphus* seed residues are reported as locally abundant, available and cheap agricultural waste in the arid and semi-arid regions where *Ziziphus mauritiana* and *Ziziphus spina-christi* are widely distributed. The regional availability is important because it is desirable to use water treatment materials that are locally available and, ideally, produced from waste biomass materials, minimizing transport and disposal costs [10,25,26]. In general, biochars and biomass-derived carbons are preferred, as they can be produced with a large surface area and porous structures and, through carbonization and activation processes, can also maintain or modify surface functionalities that are important for pollutant binding [27,28]. *Ziziphus spina-christi*, known as sidr, is a hardy native tree found across Qatar’s desert landscape, and valued for its shade, edible fruit (nabk), and traditional medicinal uses.

The performance reported for *Ziziphus*-derived materials also warrants more scrutiny. *Ziziphus* seed adsorbents have been used for dye removal and for metal removal, and recent studies indicate that oxygenated groups and aromatic domains are present on the surfaces of the adsorbents, and desirable for adsorption [10,29]. Compared with other low-cost adsorbents studied in the same research, *Z. spina-christi* seed biomass exhibited high maximum capacities for methylene blue and methyl violet, and activated carbon from jujube seed was also effective in removing Congo red [30,31]. The results cannot be generalized to all agricultural wastes, as there are some cautions related to the adsorption properties in the broader literature which indicate that they can be highly dependent on the preparation method and test conditions, and direct comparisons between different biomass classes are still limited [32]. Despite this, the available evidence suggests that *Ziziphus* seeds possess multiple desirable characteristics: they are waste-derived and locally available, can be activated and hybridized, have a desirable carbonaceous texture when processed, and are applicable to both organic and inorganic pollutants [4,33]. Another reason to study the *Ziziphus* seeds specifically is that the field has grown to a stage where more critical synthesis is needed than mere reporting of adsorption capacities. In many recent investigations, biosorbents, low-cost adsorbents, biochar, microbial sorbents, and biomass-derived carbons all present the same set of unanswered questions: limited and/or unrealistic testing of real or multicomponent wastewater, lack of regeneration and life-cycle evidence, poor engineering design analysis, and excessive reliance on laboratory batch experiments to support claims of novelty [18]. These are directly applicable to *Ziziphus*-based materials. The practical significance of these materials would require that preparation methods continue to be truly low cost, that regeneration be long-lasting, and that the performance be maintained in complex effluents, instead of the idealized single-solute systems used in the reports. This discrepancy between laboratory promise and implementation feasibility is now one of the main issues in the field of adsorption research [14,34].

The present review addresses that gap by providing a focused assessment of *Ziziphus* seed-based adsorbents for water and wastewater treatment. Unlike earlier reviews that examine adsorption in broad terms or survey large classes of biomass, biochar, microbial sorbents, hydrogels, graphene-based materials, or low-cost adsorbents, this review centers on a single under-reviewed feedstock and synthesizes its development across raw biomass, activated carbons, and modified composite systems [6,35]. The novel aspect of this review lies in its specific focus on *Ziziphus* seed-based adsorbents for water and wastewater treatment and in linking feedstock characteristics, processing routes, interfacial mechanisms, and application constraints within one critical framework, while distinguishing between well-supported findings and areas where evidence remains sparse or inconsistent. By doing so, the review critically evaluates the potential of *Ziziphus* seeds as low-cost adsorbent precursors while recognizing the current limitations related to real-wastewater validation, regeneration, scale-up, and commercial applicability.

## 2. *Ziziphus* Seed Biomass for Adsorption

The biomass of *Ziziphus* is abundant in many regions, and has many applications with varied chemical characteristics, such as raw biomass, activated carbon, biochar, magnetic biochar, and hybrid composite adsorbents, but the evidence base is limited to dye and chromium/cadmium removal and not the broad range of contaminants the field purports to address [10].

### 2.1. Botanical Characteristics

**Figure 1:**
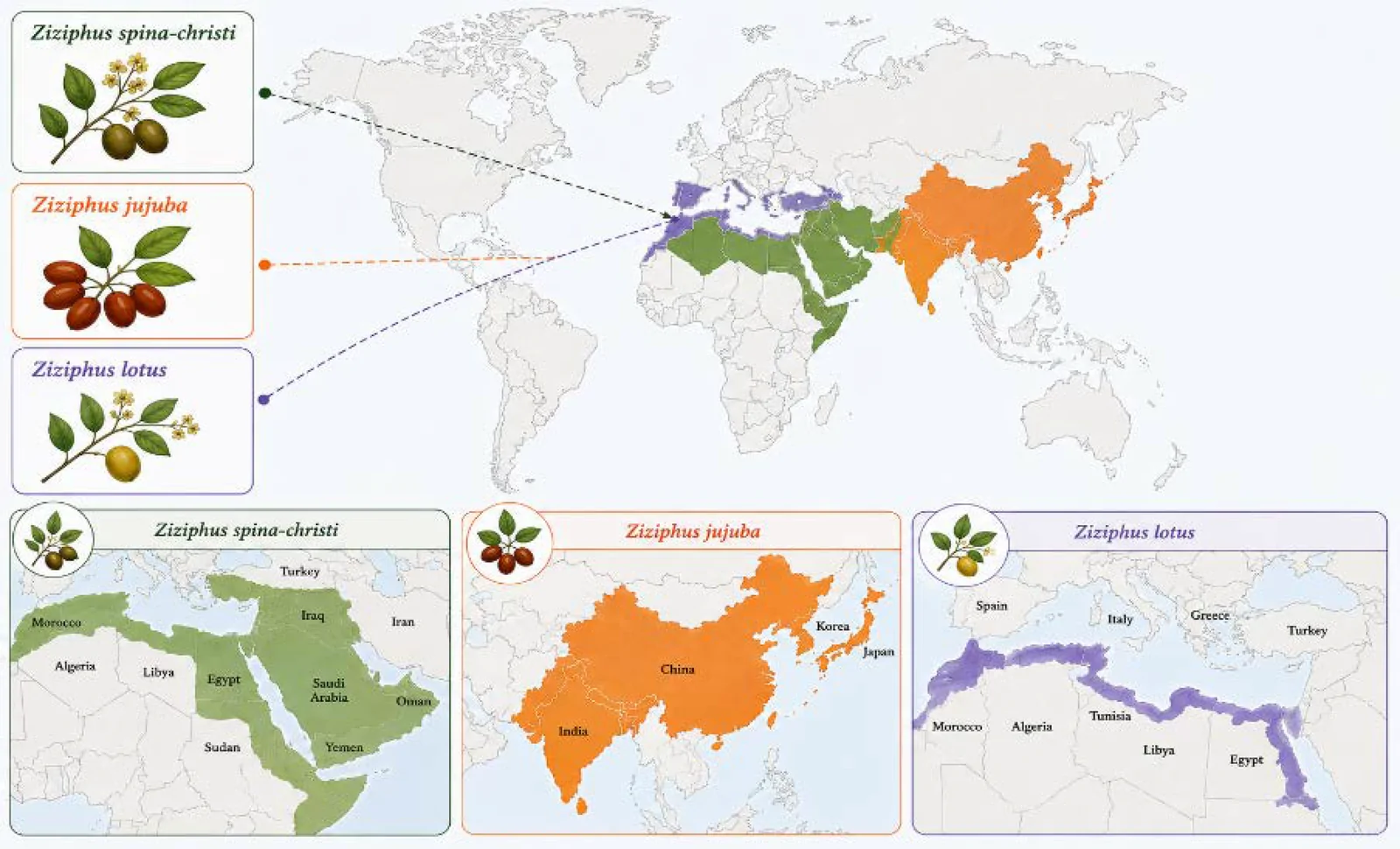
Geographical distribution of selected *Ziziphus* species.

**Figure 2:**
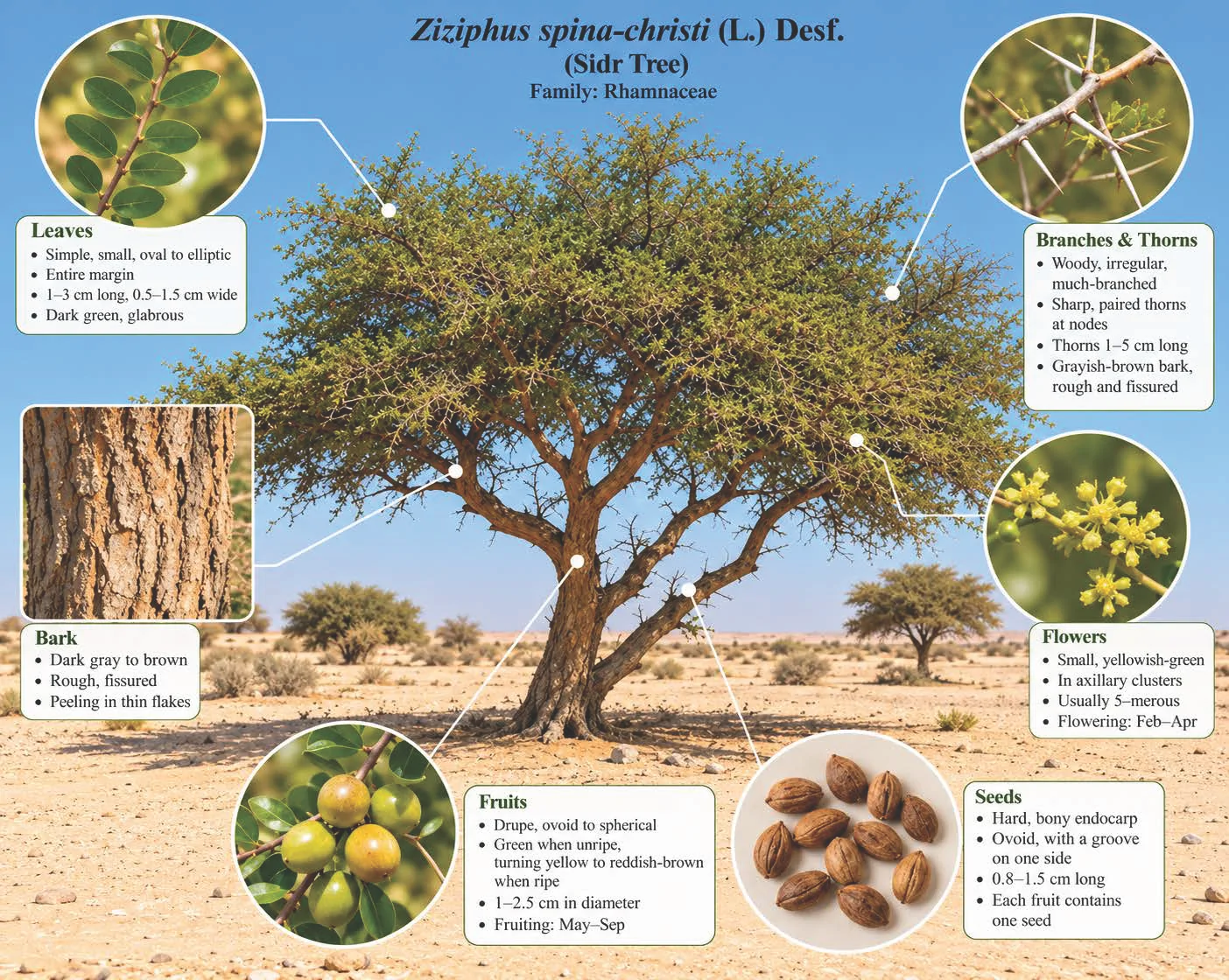
Botanical characteristics of *Ziziphus spina-christi* in Qatar.

*Ziziphus* is a genus of Rhamnaceae consisting of over 170 species, primarily from tropical and subtropical areas [23]. In the recent agronomic and botanical literature, *Z. jujuba*, *Z. mauritiana*, *Z. spina-christi*, *Z. lotus*, and *Z. nummularia* are the most economically important species for food, medicinal and biomass applications, particularly in arid and semi-arid regions of Asia, the Middle East and North Africa, as shown in Figure 1. Figure 2 illustrates the main botanical characteristics of *Ziziphus spina-christi*, including the leaves, flowers, unripe and ripe fruits, branches, and seeds. Ecological breadth is important in the production of adsorbents because feedstock security is enhanced when it is already part of the dryland farming system, rather than dependent on niche cultivation [36,37,38]. The variation in morphology of the genus is significant, with differences in stone size, fruit weight and tissue composition, and “*Ziziphus* seed” is not a single precursor, but rather a group of related biomasses, with species- and accession-level variation [37]. That variation is both a practical opportunity and a methodological issue. It provides an opportunity to choose better-quality germplasm to produce adsorbents, but it also makes it difficult to compare studies on different adsorbents (using different species, seed fractions or local landraces) without consistent reporting [39]. The availability of seed biomass is reinforced by the existence of multiple *Ziziphus* value chains that favor the use of the fruit or the medicinal parts of the plant and underutilize the seeds, stones, pulp, or related residues [36].

### 2.2. Physical Properties

The physical properties most important for adsorption include moisture content, ash content, volatile matter, fixed carbon, particle size, pore structure and thermal behavior. These control the yield of carbon, the activation response and the final balance of surface functionality and textural development [40,41]. In general, increasing the pyrolysis temperature increases fixed carbon, ash, pH and negative surface charge. 

But decreases the moisture content, volatile matter and total yield for biomass precursors [42]. There are few direct temperature-series data for *Ziziphus* specifically, but it seems to follow the same general trend. The available data on composition are limited and indicate that the seeds of *Z. mauritiana* are rich in fiber (48.12%) and carbohydrates (63.24%), while containing 1.89% fat on a dry weight basis [43]. The values are comparable to a rigid lignocellulosic precursor, which should preserve its structure during carbonization and promote pore formation during activation [44]. A consistent finding across all *Ziziphus*-derived activated carbons is that chemical activation converts this non-elastic precursor into a porous material with a large amount of accessible texture, which varies significantly depending on the precursor fraction and activating chemistry.

The results in Table 1 indicate that the performance is not determined by the identity of the feedstock. Surface area varies from approximately 100 to 846 m^2^ g^−1^ in the mentioned adsorption studies, and the surface area of carbons prepared from *Ziziphus* outside the seed-only system can reach up to 1800 m^2^ g^−1^, suggesting that the preparation method plays a significant role, in addition to the biomass source [45].

### 2.3. Chemical Composition

**Figure 3:**
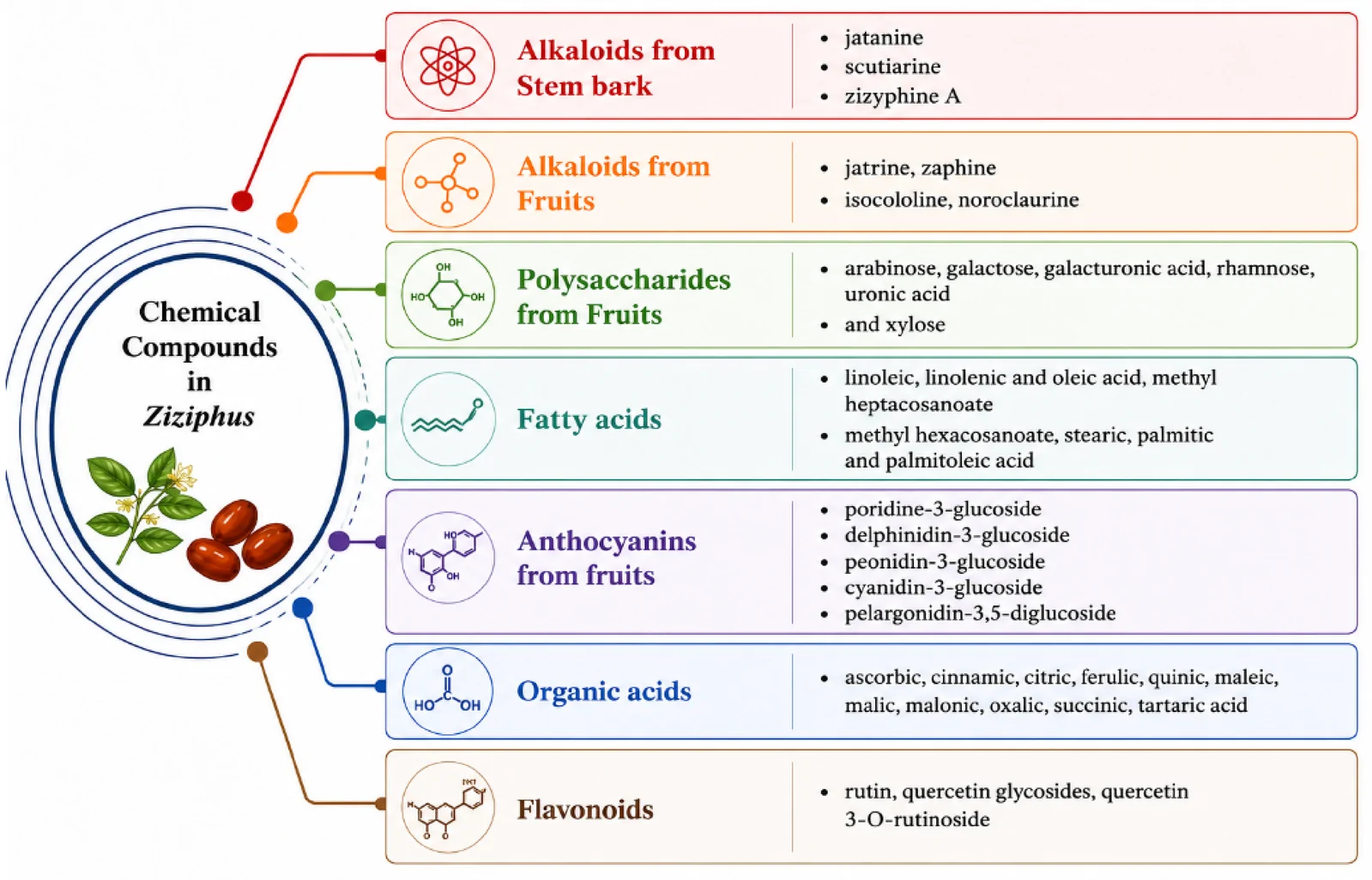
Schematic overview of the major phytochemical constituents reported in *Ziziphus*, classified according to their chemical groups and representative compounds.

The biomass of *Ziziphus* seeds should not be considered merely a carbonaceous raw material, but as a lignocellulosic raw material containing accessory lipids, proteins, minerals and extractives [30,50]. It is important because the devolatilization and formation of pores are enhanced by cellulose and hemicellulose, whereas the aromatic carbon and the higher char yield are due to the presence of lignin; the balance between these fractions has a significant impact on the carbonization behaviors and the adsorption properties of the product [51]. Although the evidence on the seeds is recent, it is not abundant; the highest crude fiber and carbohydrate contents were reported in *Z. mauritiana* seeds. A separate germplasm study revealed that seeds of *Z. jujuba* var. *spinosa* showed the highest phytochemical diversity among seed, pulp and leaf tissues; 114 compounds were identified, and there was a significant enrichment of flavonoid glycosides and saponins [43]. The presence of these phytochemicals does not mean that they are adsorbents, but rather that they are good indicators of a chemically rich precursor that can affect extractives, oxygenated groups and the chemistry of char formation. Figure 3 summarizes the principal phytochemical classes and representative compounds reported in *Ziziphus* [52]. In addition, the differences in the chemical composition of *Ziziphus* seed precursors contribute to their adsorption potential, as summarized in Table 2.

### 2.4. Surface Chemistry

Surface chemistry is a key determinant of the adsorption behavior of *Ziziphus*-derived materials. FTIR, XPS, elemental analysis, and surface-charge measurements have identified oxygen-containing functionalities and aromatic carbon domains as important surface characteristics of these adsorbents [53]. Elemental analysis (CHNS-type) and zeta potential (pH_pzc_) measurements have also been reported [49,54]. FTIR is the most frequently employed technique, as it can identify the oxygen-containing groups, which are the most common binding sites in both dye and metal systems [33]. The abundance and accessibility of these chemical features vary with precursor composition and subsequent processing. Their specific roles in pollutant binding, including electrostatic attraction, ion exchange, complexation, hydrogen bonding, and π–π interactions, are discussed in detail in Section 5 [29,55]. One of the most important advantages of some *Ziziphus* carbons is that they can be activated to generate microporosity while at the same time retaining acidic surfaces or oxygen-rich surfaces. For example, one adsorbent had an 846 m^2^/g surface area, which is good for the uptake of cationic dyes, but not as good for the uptake of anionic dyes unless the solution pH is carefully adjusted [47]. In contrast, the KOH modification of *Z. spina-christi* stones was more effective at removing heavy metals because of the increase in carboxylate ions, indicating that chemical tuning of charge can be more important than maximizing surface area [56,57].

### 2.5. Advantages of Ziziphus over Other Wastes

The biomass of *Ziziphus* seeds is comparable to common agricultural wastes, but not always better. Coconut shell remains a benchmark precursor, as it shows high fixed carbon and well-developed porosity. One recent comparative study showed that coconut shell achieved the highest methylene blue removal among the five wastes tested, with a BET area of 1000.15 m^2^ g^−1^ [58]. The seed activated carbon (SAC) can be used to remove methylene blue, with an adsorption capacity of 165 mg g^−1^, although this capacity varied considerably among seed types and was lower than that of commercial activated carbon in produced water treatment [59]. In contrast, the walnut shell is highly desirable for its low ash content, chemical stability and potential for large surface area [60].

It can be observed from Table 3 that the comparative advantage of *Ziziphus* is not just the absolute capacity. Its advantage lies in its local availability in dry areas, the tunability of its chemical properties, and its versatility across activated carbon, magnetic biochar, and hybrid composite formats [61].

### 2.6. Ziziphus-Based Adsorption Studies 2020–2025

**Figure 4:**
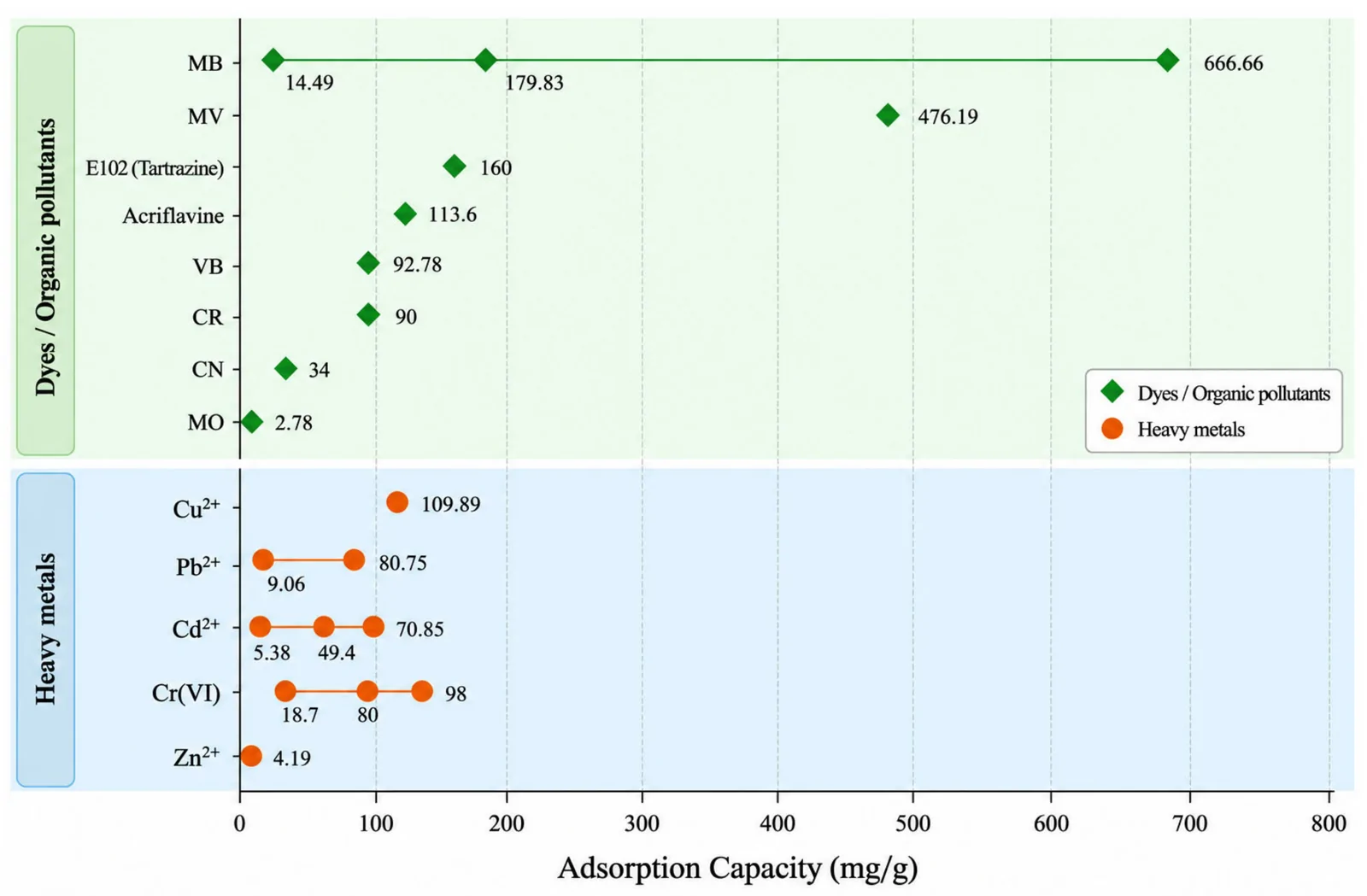
Reported adsorption capacities for different pollutants using *Ziziphus* seed-based adsorbents (2020–2025) [10,29,32,46,47,48,54,61].

The extensive research between 2020 and 2025 has been dominated by dyes and heavy metals, while there is only scattered research on phenolics or catalytic degradation of dyes, and no solid evidence has been found so far for pharmaceuticals, pesticides, nutrients, fluoride or oil [64]. Furthermore, Table 4 presents a summary of representative studies evaluating the adsorption performance of *Ziziphus* seed-derived materials. This mismatch is important because many review articles refer to the emerging contaminants as a target class, but the *Ziziphus* seed literature has not yet been able to support that. A bubble plot in Figure 4 was constructed from the *Ziziphus* seed-based adsorption studies reviewed for the period 2020–2025, with the reported adsorption performance grouped according to pollutant type and adsorbent system.

**Figure 5:**
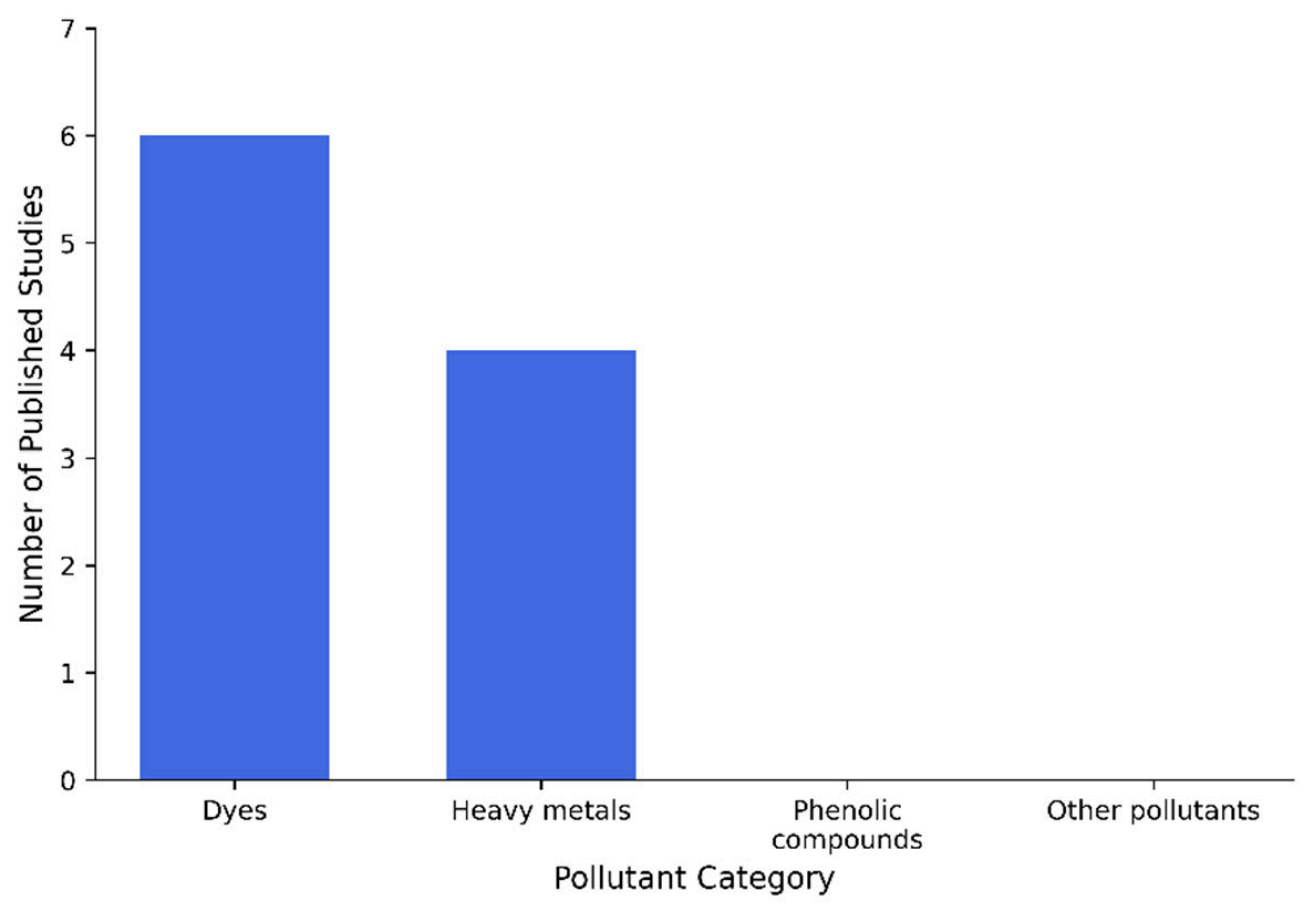
Distribution of published studies on *Ziziphus* seed-based adsorbents by target pollutant category (2020–2025).

Figure 5 summarizes the distribution of pollutant categories investigated in *Ziziphus* seed-based adsorption studies published between 2020 and 2025. Among the studies included in this review, dyes were the most frequently investigated pollutants (*n* = 6), followed by heavy metals (*n* = 4), whereas no seed-based studies were identified for phenolic compounds, pharmaceuticals, pesticides, nutrients, fluoride, oil, or other emerging contaminants. The distribution reveals a definite research focus on color removal and toxic metals, with very little attention paid to the contaminant classes most important for advanced wastewater treatment [23]. Recent studies have focused on modifying *Ziziphus* seed-derived adsorbents to improve porosity, surface functionality, and adsorption capacity [46]. The key question is whether that laboratory versatility will translate into standardized, economically viable performance in the more common multicomponent wastewaters, or remain confined mostly to single-solute batch systems [41].

## 3. Preparation of *Ziziphus* Seed Adsorbents

The adsorption properties of *Ziziphus* seed adsorbents depend primarily on the way the seeds are cleaned, sized, carbonized, activated, and surface-modified, as each of these processes alters the porosity, surface chemistry and recoverability [65]. Overall, the studies available on *Ziziphus*-based adsorbents clearly show that simple raw biomass can already be used for the removal of dyes and metals, but that the potential benefit is typically achieved by chemical activation or targeted hybridization, which in turn increases the amount of reagent required, the washing load, and the complexity of scale-up [46].

### 3.1. Raw Biomass Preparation

The preparation of raw biomass is methodologically straightforward; however, careful control of the washing, drying, grinding, and sieving steps is essential because each stage influences the physicochemical properties of the resulting adsorbent. Washing removes adhering dust, soil particles, and water-soluble impurities, thereby reducing inorganic contaminants and modifying the ash content and surface composition. Drying to a constant mass minimizes variability caused by residual moisture, ensuring consistent adsorbent dosage and improving the reproducibility of adsorption experiments. Grinding and sieving produce a more uniform particle size distribution, increasing the external surface area, shortening diffusion pathways, and enhancing the accessibility of adsorption sites. Consequently, variations in these preparation steps may alter adsorption capacity, apparent adsorption kinetics, and batch-to-batch reproducibility of the raw biosorbent [66]. Recent studies that employed *Ziziphus* seeds or stones typically employed a similar basic methodology, utilizing repeated washing to remove dirt and soluble impurities, drying to constant mass in the oven, and crushing or milling to yield a raw sorbent or a precursor for carbonization and activation [67]. This simple pretreatment is consistent with conventional biosorbent processing, where washing, drying, and particle size reduction represent the standard initial steps before direct adsorption experiments or further thermochemical modification [68]. A major practical advantage of this preparation strategy is that it avoids energy-intensive carbonization and chemical activation, thereby reducing processing complexity and material preparation costs. Despite the absence of these additional modification steps, a raw *Ziziphus spina-christi* seed biosorbent prepared only by washing, drying, grinding, and sieving exhibited remarkably high Langmuir maximum adsorption capacities of 666.66 mg g^−1^ for methylene blue and 6.19 mg g^−1^ for methyl violet [10]. However, these exceptional adsorption capacities were reported for cationic dyes and should not be generalized to other contaminant classes, particularly heavy metals and larger organic molecules, whose adsorption mechanisms and pore accessibility requirements differ substantially [65,69]. Standardization is another drawback. Although these factors may influence diffusion rates, apparent kinetics, and mass-transfer interpretation, moisture normalization, particle size cutoffs, and storage conditions are rarely reported in the current *Ziziphus* literature. Raw biomass routes are also attractive for rural or decentralized treatment, but they are also the least well-developed in terms of quality control, since the raw biomass is directly transferred to the sorbent [70,71].

### 3.2. Carbonization

**Figure 6:**
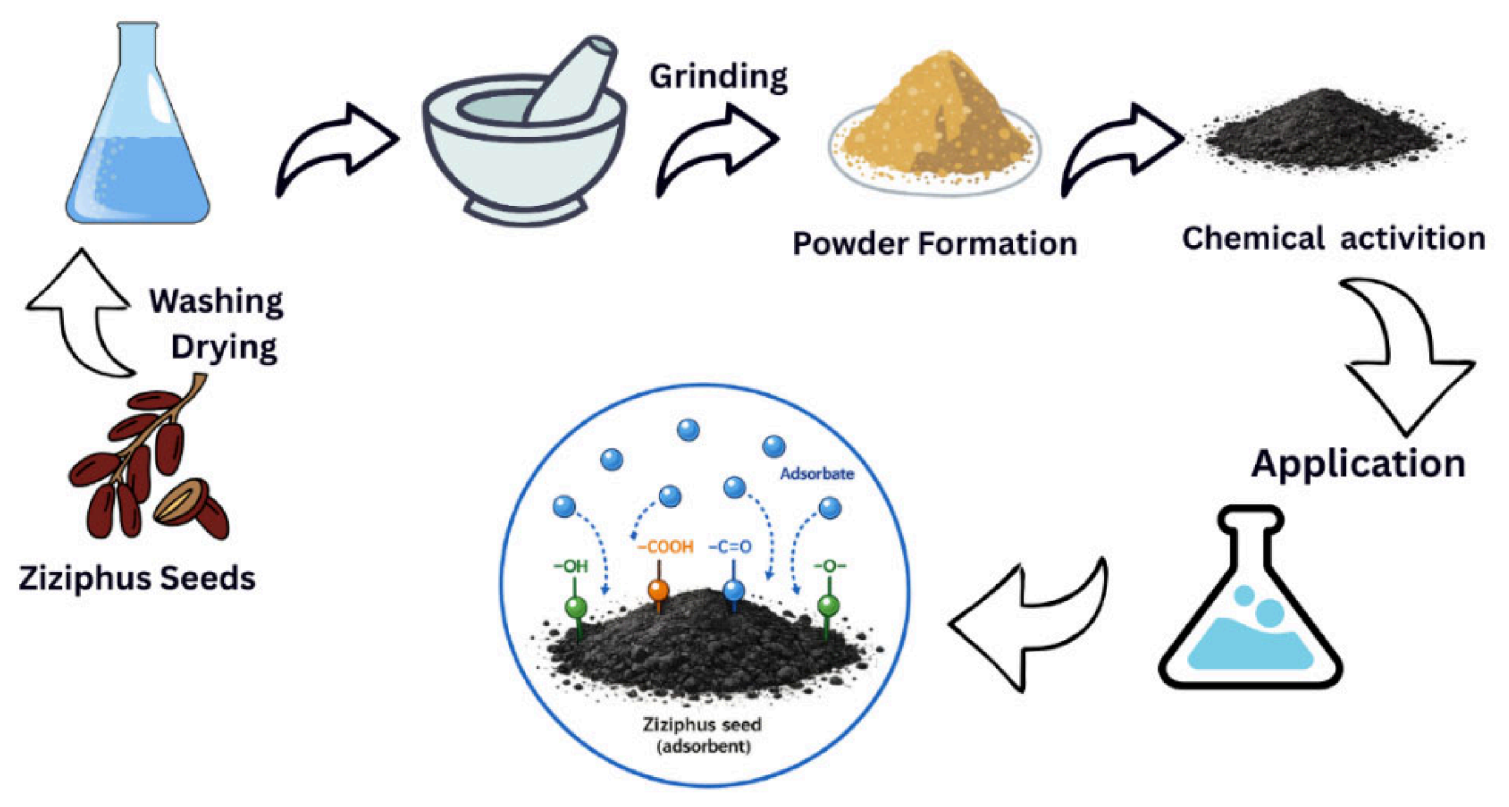
Synthesis workflow detailing the extraction and carbonization of *Ziziphus* seeds for adsorption applications.

The first significant structure-forming step is carbonization, which transforms the lignocellulosic seed matrix into a carbon-rich solid that is characterized by its pore network, aromaticity and residual functional groups, which are highly sensitive to the temperature, heating rate, residence time and atmosphere [70,72]. As an example, Figure 6 shows the steps of carbonization in a type of *Ziziphus* seed. The overall literature on biochar is consistent in that moderate temperatures between 400 and 700 °C are generally found to be good for developing the pores, while temperatures that are too low result in insufficient carbonization, and temperatures that are too high result in burn-off, loss of some structure or loss of surface functionality [65]. For *Ziziphus*-derived carbons, temperatures around 500 °C have frequently produced well-developed carbonaceous structures; however, the final textural properties also depend strongly on subsequent activation conditions [46,48,73]. In many cases, such as phenol, methylene blue and CO_2_ analog systems, the porous structure is more important than the functional groups, making the untreated biochar less effective than activated carbons [74]. This is why most high-performing *Ziziphus* adsorbents do not stop at pyrolysis. An open question remains: while factorial studies on other systems of biomass have indicated that temperature, residence time, and particle size are highly coupled and can vary surface area by orders of magnitude, few *Ziziphus* studies have optimized all three parameters simultaneously [75].

### 3.3. Activation Methods

The central upgrading step is activation, which determines how much of the latent pore structure formed during carbonization becomes accessible and which surface chemistry is available for adsorption.

#### 3.3.1. Physical Activation

Typically, biochar is physically activated by exposing it to CO_2_, steam, air, ozone, microwave heating, ultrasound, plasma, or other oxidizing environments to deepen and open pores without adding chemical salts or acids [76,77]. The major benefit is cleaner production and easier post-treatment, but the drawback is that the resulting carbons are generally less porous than those obtained by chemical activation [78]. The direct physical activation studies on *Ziziphus* seeds are still few and far between, and this is a research gap. No research paper in the existing *Ziziphus* corpus offers a well-developed steam- or CO_2_-only activation workflow similar to the acid-activation literature [45]. The absence is significant because steam and CO_2_ routes are also industrially attractive. In non-*Ziziphus* systems, steam tends to widen pores and increase mesoporosity, while CO_2_ has a stronger effect in creating microporosity; in some cases, mixed atmospheres can be synergistic, but in others, they are not [79]. Another step, pre-oxidation, can enhance physical activation by generating more pores and increasing biochar reactivity [80]. For *Ziziphus*, it is an untested opportunity: physical activation may decrease the effluent and neutralization load of the acid washing process, but it has yet to be compared with the H_3_PO_4_ and H_2_SO_4_ routes. The gap is significant for industrial translation, as large-scale water treatment will select for processes with fewer reagents, less saline wash water, and fewer hazardous regeneration streams.

#### 3.3.2. Chemical Activation

Chemical activation is more widely reported in the *Ziziphus* literature, as it produces more surface area and better pore development and surface functionality compared to physical treatment and is more effective at lower temperatures [81]. It plays a crucial role in tailoring the physicochemical properties and adsorption performance of *Ziziphus* seed-derived materials, as presented in Table 5. The most convincing evidence concerns H_3_PO_4_, H_2_SO_4_, ZnCl_2_, and alkali treatments; however, they are not equally effective. For dye-oriented *Ziziphus* carbons, H_3_PO_4_ is the most successful activator. It generated a mesoporous Indian jujube seed carbon with a surface area of 571 m^2^ g^−1^ for the removal of acriflavine and Victoria blue B [46]. It also produced a highly mesoporous *Z. spina-christi* carbon with a surface area of 846 m^2^ g^−1^ and excellent tartrazine uptake, with an impregnation ratio of 2:1 and a carbonization temperature of 500 °C [47]. Nevertheless, the adsorption capacity of H_3_PO_4_-activated *Z. jujuba stones* is not always the highest: in the case of a reactive commercial dye, the BET area is very high (1896 m^2^ g^−1^), but the adsorption capacity is low (37.04 mg g^−1^), suggesting that accessible chemistry, pore size matching and solution speciation can outweigh surface area [82]. H_2_SO_4_ activation seems to be more variable. Conversely, the malachite green capacity of *Z. spina-christi* shell seed adsorbent was enhanced from 48.78 to 370.37 mg g^−1^ after sulfuric acid treatment, which is a substantial increase, and this may be attributed to the opening of pores and the introduction of acidic functionalities [66]. However, the maximum adsorption capacity of the porous carbons produced from the cores of *Z. lotus* by using sulfuric acid was still moderate at 14.493 mg g^−1^ and the BET area was high, emphasizing that a mere increase in textural properties is not sufficient to obtain better adsorption properties [48]. ZnCl_2_ is particularly useful for metal-oriented sorbents and for increasing the microporosity of the wider biomass systems. The activation using ZnCl_2_ resulted in a heterogeneous Cd^2+^ sorbent of *Ziziphus jujuba* seed-derived biomass with a capacity of 49.40 mg g^−1^ and stable performance for 4 cycles [32]. The ZnCl_2_ activation has been shown to enhance the surface area and micropore volume, especially near 500 °C, which is consistent with its mechanistic plausibility for seed-based carbons as well [83]. The disadvantages are that it requires extensive downstream washing, may produce effluents containing chloride, and has a greater regulatory burden than purely thermal routes. In addition, alkali treatments are more often used in biosorption-based modification than in total production of activated carbon; however, they may play a critical role in the binding of metals. KOH-modified *Z. spina-christi* stones were found to be more effective in the removal of Pb^2+^, Zn^2+^ and Cd^2+^ compared to the acidic modifications, due to the increases in carboxylate and alcohol groups and the unblocking of binding sites [29]. This is significant because the finding demonstrates that the “best activation” is pollutant-specific: acidic routes will be good for the uptake of dyes, while alkaline routes will be good for the exposure of oxygen sites with anionic character, which are necessary for metal complexation.

### 3.4. Surface Modification

Beyond the activation of surfaces in *Ziziphus* systems, new hybrid adsorbents with magnetic separability, catalytic activity, or new coordination sites have been developed. The key conceptual change is from maximizing surface area to engineering multifunctionality. A physically dominated spontaneous and exothermic adsorption process on a heterogeneous surface was used to enhance the uptake of hazardous dyes, using graphene oxide–*Ziziphus* composites [10,84]. In one study, the magnetic biochar derived from *Ziziphus jujuba* seeds improved recoverability and achieved high chromium removal (98% in batch and 99% in column operation) and >95% desorption under HCl [54,85]. Another approach was taken by the Zn-loaded *Ziziphus mauritiana* seed activated carbon, which combined adsorption with photocatalysis by ZnO-supported carbon, instead of just using adsorption [54]. Such modifications improve usability but make scale-up more difficult. In principle, magnetic composites could lower the separation energy and enhance the process handling [86], but they also add additional synthesis steps, inorganic reagents, and possible leaching and/or stability issues that have not been addressed directly in the current *Ziziphus* literature. The same applies to photocatalytic hybrids: they can expand functionality, but their economic argument can be less convincing if the added material is not significantly superior to the simpler activated carbons under actual effluent conditions. Table 6 summarizes representative structural modification strategies for *Ziziphus*-derived adsorbents, highlighting their functional advantages, adsorption performance, and associated limitations.

### 3.5. Scale-Up Implications

The preparation literature suggests that the best-performing *Ziziphus* adsorbents are generally the most complicated to prepare. Although H_3_PO_4_- and ZnCl_2_-based activation can generate highly developed porous structures, these routes require careful control of impregnation, extensive post-activation washing, and management of chemical-containing liquid waste [23]. In contrast, minimally processed *Ziziphus* seed biomass may offer a simpler and potentially more scalable route by avoiding complex activation and modification procedures. For example, *Z. jujuba* seed-derived biomass has been successfully applied as a cost-effective biosorbent for Cr(VI) removal [87]. However, further studies on continuous operation, regeneration, and techno-economic feasibility are required to assess its large-scale applicability [88]. The field therefore still lacks a robust techno-economic comparison between raw biomass, acid-activated carbons, alkali-modified sorbents, and magnetic composites made from the same *Ziziphus* precursor. That missing comparison is now more important than adding another single-pollutant batch study. The most important shift in this literature is the transition from using *Ziziphus* seeds as a simple agricultural waste to engineering them into functional adsorbents.

## 4. Characterization of *Ziziphus* Seed-Derived Adsorbents

Characterization of adsorbents prepared from *Ziziphus* seeds has three interrelated goals: verification of the accessibility of the porosity created during preparation, identification of the surface chemistry responsible for the binding of pollutants, and elucidation of the reasons for the differences in performance among raw biomass, activated carbons and modified composites. The most informative data in the *Ziziphus* literature are provided by a combination of microscopy and spectroscopy with textural data, since none of these techniques alone can reveal whether pore development, oxygenated functionalities, mineral phases, or surface charge dominates adsorption [89].

### 4.1. Morphology and Texture Properties

**Figure 7:**
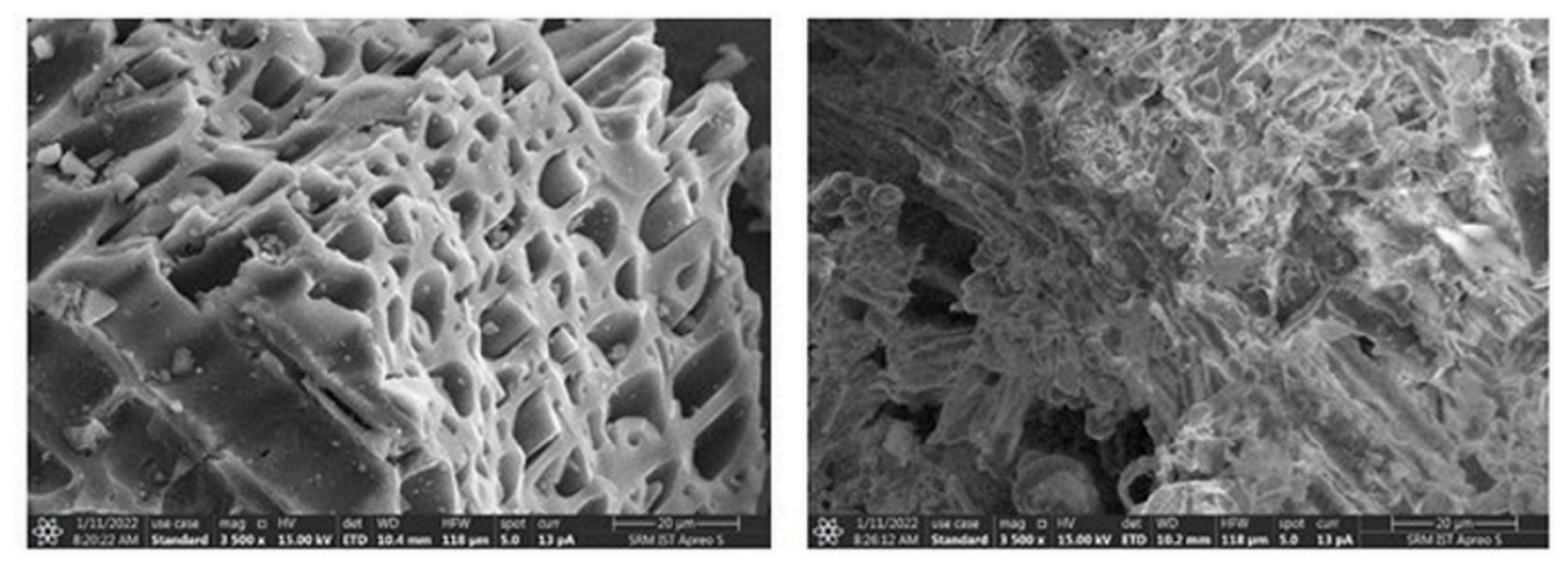
High-resolution SEM micrographs of *Ziziphus* seed surface morphology before and after adsorption [88].

Scanning electron microscopy (SEM) is used to observe the surface roughness and pore development, and it is consistently found that activation transforms relatively compact biomass into fractured, porous solids [88]. For example, sulfuric-acid-treated *Z. lotus* carbon exhibited pores of approximately 10–45 μm, while related materials investigated for Cd and Cr(VI) removal showed pores of approximately 6–50 μm [49,88]. These observations show a correlation between the open morphology and rapid uptake, as shown in Figure 7. BET analysis complements these morphological observations by quantitatively determining specific surface area, pore volume, and pore-size distribution [90]. The BET surface area of the *Ziziphus*-derived activated carbons ranges from 1 to 970 m^2^/g, indicating that the preparation method affects the texture more than the feedstock does [91]. This is important because wider carbon studies indicate that a higher microporosity and larger surface area are generally associated with better adsorption, provided that the pores are not too small to be accessed by the specific pollutant under study [92,93].

### 4.2. Surface Chemistry and Structure Properties

The oxygen-containing functional groups that frequently participate in metal complexation and dye binding have been identified in the *Ziziphus* literature, and Fourier transform infrared (FTIR) spectroscopy is the most consistently used method [88]. Activated carbon from *Z. lotus* and *Z. jujuba*, in addition to ZnCl_2_-activated *Ziziphus* biomass, was used in the interpretation of the mechanisms of Cd^2+^ and Cr(VI) uptake, respectively, using FTIR [33]. In general, mechanism assignments using FTIR for modified biochar tend to consistently point to hydrogen bonding, electrostatic attraction, and π–π interactions for dyes, and oxygen-group complexation for metals [31]. Moreover, XRD is used mainly to distinguish amorphous carbon from crystalline mineral phases. The broad peaks around 2θ ≈ 25° in the activated carbon of *Ziziphus lotus* suggested that it was mainly amorphous carbon, and the small peaks were related to ash. That is the typical result for biomass carbons, and a more amorphous character is typically correlated with better defect chemistry and adsorption reactivity than highly ordered graphitic structures. Another point related to the Raman data from GO-*Ziziphus* and non-*Ziziphus* activated biochar is that the defect-rich sp^2^/sp^3^ carbon organization changes with the severity of the activation and can follow the performance differences that BET is not able to detect [89,94]. XPS, on the contrary, is underutilized in the studies of *Ziziphus*, but is the most powerful technique for the assignment of the elemental states on surfaces and the redox changes after adsorption. For analogous modified biochar, XPS results indicated that both physical adsorption and chemical reduction occurred during Cr removal, and the N- and O-containing groups in the modified biochar were involved in the Cr conversion process [95,96]. The information obtained from SEM and BET, however, is not reliable enough to extract information on metal adsorption and therefore, future investigations of metal adsorption on *Ziziphus* should include routine pre/post-adsorption XPS in addition to FTIR.

### 4.3. Charge, Composition, and Technique Roles

EDX is typically employed with SEM to verify the elemental composition and post-adsorption loading, but not to provide detailed information about the surface chemistry [90]. For activated *Ziziphus jujuba seeds*, EDX was specifically used to confirm the presence of hexavalent chromium after uptake, and for *Ziziphus* biomass activated with ZnCl_2_, SEM-EDX was used in combination with FTIR and XRD to evaluate the physicochemical properties relevant to Cd^2+^ adsorption [88]. The structural evolution of *Ziziphus* seed-derived materials following modification can be effectively monitored by X-ray diffraction (XRD). The property that is not measured by most of the structural techniques is the surface charge as a function of pH, which is measured by zeta potential and pH_pzc_ measurements. The pH_pzc_ of *Z. spina-christi* activated carbon was 4.03, which is acidic enough to allow cationic adsorption to take place at a pH above 4.03, while at pH values below 4.03 the surface becomes positively charged, which can favor anionic adsorption, depending on other interactions. More comprehensive modified-biochar experiments have demonstrated that acid protonation can enhance both the electrostatic attraction and nitrate adsorption, as well as the zeta potential and pore volume of biochar, while base deprotonation can reduce these effects [95,97]. The lesson to be learned from *Ziziphus* adsorbents is that, when comparing metals, cationic dyes, anionic dyes, and oxyanions, it is important to characterize the charge.

The current *Ziziphus* characterization literature is not scarce in techniques, but rather in integrated interpretation. Many studies have reported SEM, FTIR, and occasionally XRD, with fewer combining these with the XPS, zeta potential, and thorough BET analysis that clearly differentiate between pore-controlled adsorption and charge-controlled or redox-controlled adsorption, as summarized in Table 7. The next section, then, should address the connections that can be made between these characterization results and the pathways of adsorption, since characterization is only useful if it predicts the behavior of pollutants rather than just describing the material.

## 5. Adsorption Mechanisms of *Ziziphus* Seed Adsorbents

The adsorption mechanisms of *Ziziphus* seed-derived adsorbents are crucial, since the performance is not only dependent on the surface area but also on the interactions between the surface chemistry induced by the preparation procedure, the pollutant speciation and the operating conditions, like pH, ionic strength, and temperature. In the *Ziziphus* literature and the general biomass-carbon literature, adsorption is not typically dominated by a single pathway, but is instead a combination of electrostatic attraction, pore filling, hydrogen bonding, ion exchange, surface complexation, and π–π interactions, with the primary contribution varying among contaminant classes and material design [98,99]. The dominant adsorption pathways and their controlling variables are summarized in Table 8.

### 5.1. Electrostatic and Exchange Effects

Electrostatic attraction is the most condition-sensitive mechanism, since it relies on pH_pzc_, solution pH and pollutant speciation, which are not solely dependent on intrinsic carbon texture [99]. The adsorption of the cationic dyes was found to increase with the increase in pH; the *Ziziphus* seed carbons exhibited the highest adsorption of acriflavine and Victoria blue B at pH 8–10, which is similar to the raw *Z. spina-christi* seed biomass, which also performed best in the near neutral range at pH 8–10 for the adsorption of methylene blue and methyl violet [103]. In contrast, anion removal can shift the optimum towards acidic conditions, as illustrated by the uptake of Cr(VI) on *Ziziphus jujuba*-derived carbons, where the optimum removal was at around pH 2, due to the preference of chromate adsorption on protonated surfaces [104]. However, electrostatics is not always the dominant mechanism, and this is one of the most critical points. In a recent biochar study, maximum removal of methylene blue was observed at pH 4, although the biochar had a high pH_pzc_, due to the diminished repulsion caused by the near-neutral zeta potential and the increased strength of hydrogen bonding and π–π interactions [105]. The same can be said for *Ziziphus* systems with a significant amount of dye uptake over a wide range of pH or where the effects of pH are less than what would be predicted from pH_pzc_ [104]. In the case of dyes, exchangeable cations or ionizable acidic groups on the carbon surface are not as important as in the case of inorganic pollutants. In heavy-metal systems, biochar-bound Ca^2+^, Mg^2+^, K^+^ and H^+^ may be displaced by dissolved metal ions and this is often in addition to surface complexation and precipitation [106]. For Cr(VI), ion exchange may follow reduction to Cr(III); in one mechanistic study, the amount of Cr(VI) taken up by ion exchange was small compared to the amount taken up by redox, and the reduction was included in the calculation [107].

### 5.2. Complexation and Aromatic Interactions

Surface complexation is the most promising mechanism for the removal of heavy metals by *Ziziphus*-based adsorbents, since oxygenated groups are always present, and similar carbons are known to coordinate metals in a metal-specific manner through sites containing –OH and –COOH, as well as N- and P-containing sites [108]. The general trend of the broad carbon-adsorbent evidence is that the mechanisms of heavy-metal uptake are typically chemisorptive processes including surface complexation, ion exchange, and precipitation, and not just physisorption [109]. This is particularly evident in the case of Pb(II), which has lower binding energy and stronger affinity for oxygenated groups and therefore binds more strongly than Zn(II) and Cd(II) [110]. This is where spectroscopic evidence comes into play. FTIR peak shifts following adsorption are generally indicative of hydroxyl, carbonyl, and carboxyl functionality, whereas XPS is more directly indicative of changes in metal–ligand coordination and oxidation state [111]. In modified biochar, N- and P-containing groups provide more complex coordination environments for the removal of Pb(II); in chitosan- or amine-modified carbons, selective enhancement for Cu(II) or Pb(II) has been observed, highlighting that modification with heteroatoms can not only increase affinity but also alter selectivity [111]. Unlike metals, π–π interaction is more significant for aromatic organic pollutants and increases with the increasing graphitic or conjugated domains during carbonization and activation processes [112]. The adsorption of acriflavine, Victoria blue B and GO–*Ziziphus* dye on *Ziziphus* seed were all explained with the help of π–π interactions between the aromatic dye rings and aromatic carbon domains [46]. The same is reported for malachite green and methylene blue on non-*Ziziphus* carbons, although the strength and direction of π–π electron donor–acceptor interactions are found to depend on whether the carbon surface is rich in electron-donating hydroxyl groups or electron-withdrawing carbonyl groups [113].

### 5.3. Pore Filling and Adsorption Behavior

High performance requires pore filling, but that is not the only explanation. Pore filling contributes to adsorption when the pore dimensions of *Ziziphus*-derived carbons are accessible to the target adsorbate. As discussed in Section 3.3 and Section 4.1, activation can substantially increase the accessible porosity of these materials. However, adsorption capacity does not scale directly with BET surface area, because pore-size compatibility, surface charge, and functional-group chemistry also determine adsorbate accessibility and affinity [45,114,115]. Indeed, a large surface area does not always ensure better adsorption. The activated carbon of *Ziziphus lotus* exhibited a large surface area and porous structure but low methylene blue capacity, suggesting that the total BET area may not be the most important parameter for determining the capacity of the activated carbon, as the pore accessibility, surface charge, and identity of functional groups may also play a role [47]. Similarly, the smaller pore size, even if chemistry is favorable, has limited phenol adsorption on different biochars, and the ability to adsorb pharmaceuticals can be low if the oxygenated functionality or ionization compatibility is not good on the carbonaceous surface [99,116]. From a practical standpoint, it is important to note that pore-size matching of *Ziziphus* adsorbents should be based on the specific pollutant to be removed and not based solely on maximizing the BET area.

### 5.4. Chemisorption and Physisorption

**Figure 8:**
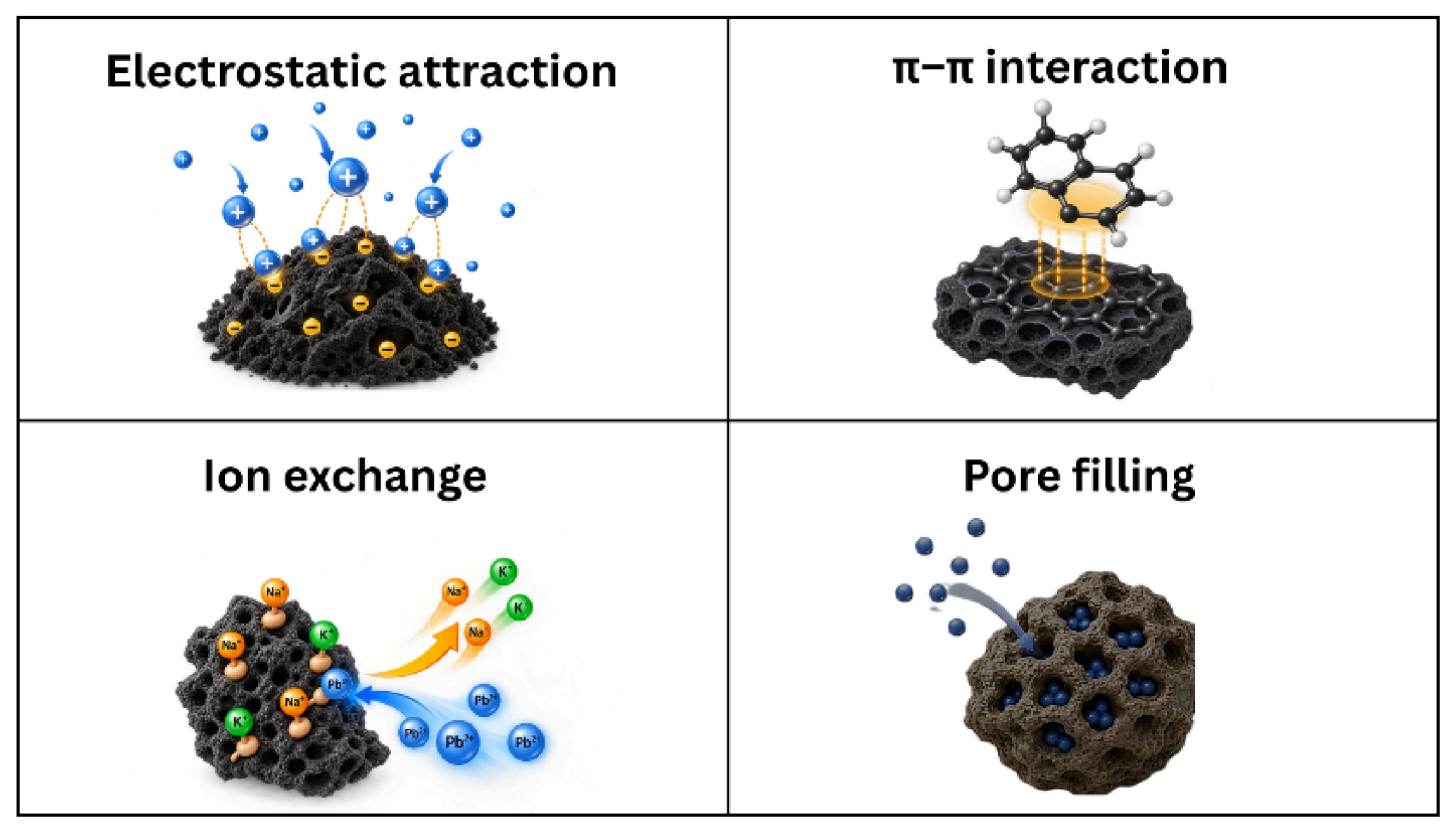
Mechanistic illustration of various interactions mediating the physical capture of pollutants on the *Ziziphus* adsorbent surface. In the electrostatic attraction panel, blue (+) spheres denote cationic pollutant species and yellow (−) symbols denote negatively charged surface sites; in the ion exchange panel, orange and green spheres denote exchangeable Na^+^ and K^+^ ions released from the surface, and blue spheres denote incoming Pb^2+^ ions; in the pore filling panel, dark blue spheres denote pollutant molecules. Arrows indicate the direction of ion/molecule migration.

The distinction between chemi- and physisorption is more of a continuum than a dichotomy. Many *Ziziphus* studies report pseudo-second-order kinetics, but this does not necessarily mean that the mechanism is purely chemical; it simply means that the rate behavior is consistent with site-controlled uptake [25]. The presence of FTIR/XPS changes, high enthalpy or activation energy, selective coordination, redox conversion or limited reversibility provides stronger evidence for chemisorption [117]. For heavy metals and Cr(VI), the combination of redox, inner-sphere complexation, ion exchange, and precipitation is most likely to occur [107]. In contrast, dye and pharmaceutical adsorption exhibits mixed behavior. In the case of GO–*Ziziphus* dye adsorption, adsorption was spontaneous, and it was explicitly interpreted as physical adsorption, while phosphoric-acid-activated jujube seed carbon was best described by hydrogen bonding, electrostatic attraction, and π–π interactions, which are largely non-covalent [10]. Other biomass carbons display the same ambiguity: some have been reported to exhibit pseudo-second-order kinetics and thermodynamic signatures of physisorption, which suggests that rate law and bonding mode should not be equated [98,118]. These systems contain van der Waals forces, hydrogen bonding, hydrophobic partitioning and weak π-stacking, which are all forms of physisorption that are particularly significant for aromatic dyes, phenolics and pharmaceuticals [100]. The principal physical interactions involved in pollutant adsorption onto *Ziziphus*-derived adsorbents are illustrated in Figure 8 [100]. Interestingly, for other biochars, hydrogen bonding can be dominant when electrostatics are weak, such as in methylene blue and sulphapyridine [119,120]. For *Ziziphus*-derived materials, this likely explains why adsorption can remain high even when charge-based predictions alone would suggest weaker affinity.

### 5.5. Integrated Mechanistic Synthesis

The dominant adsorption mechanism depends on the combined effects of pollutant class, surface chemistry, and adsorbent modification. For heavy metals, *Ziziphus*-based adsorbents commonly involve surface complexation, ion exchange, electrostatic attraction, and, in some systems, redox or precipitation processes, whereas aromatic dye adsorption is mainly associated with electrostatic attraction, hydrogen bonding, π–π interactions, and pore filling [121]. The general carbon literature suggests that in the case of pharmaceuticals and other emerging contaminants, π–π interaction, hydrophobicity, hydrogen bonding and pH-dependent electrostatics are all likely to be involved in controlling the adsorption, but this has been little tested for *Ziziphus* seed adsorbents specifically [112]. Surface modification can shift the relative contribution of individual adsorption mechanisms by altering surface charge, functional-group density, pore accessibility, and the availability of coordination-active sites [114]. The most common gaps in the literature are the lack of adequate pre/post adsorption spectroscopy, particularly XPS; mapping of zeta-potential as a function of pH; and competitive adsorption experiments [122]. In real multicomponent effluents, dye–metal competition, pore blockage, ion screening, and complex formation may alter adsorption capacity and selectivity [123]. Addressing these effects requires competitive adsorption studies using multicomponent real wastewater, supported by pre- and post-adsorption characterization to identify changes in surface chemistry and active sites. Such studies would clarify how coupled adsorption mechanisms operate under realistic conditions and improve the practical evaluation of *Ziziphus* seed-derived adsorbents.

## 6. Adsorption Factors in *Ziziphus* Seed Systems

The efficiency of adsorption onto the adsorbents prepared from *Ziziphus* seeds is dependent on the combined effect of solution chemistry, adsorbent structure and pollutant speciation and not on any single operating parameter. In the adsorption-specific literature, all of these factors (pH, contact time, dosage, initial concentration, and temperature) are found to affect performance [121]. However, *Ziziphus* adsorbents are also dependent on preparation-dependent properties like pH_pzc_, pore architecture, and surface functional-group density [124]. Based on the collected studies, several common operating conditions significantly influence the adsorption efficiency of *Ziziphus* seed-based adsorbents. These key parameters and their reported effects are summarized in Table 9.

### 6.1. Solution Chemistry

The most important operating parameter is pH, as it affects both the surface charge of the adsorbent and the speciation of the pollutants. The pH_pzc_ of *Ziziphus*-based carbons ranges from acidic to basic, e.g., *Ziziphus jujuba* seed carbon for cationic dye adsorption has its optimum pH range at 8–10, while *Ziziphus jujuba* core carbon for Cr(VI) adsorption has its optimum pH at 2 due to the protonated surface for the uptake of anions [100]. The same charge logic can be seen in the wider range of biomass adsorbents, where cationic methylene blue is better adsorbed at pH values greater than pH_pzc_, and anionic dyes like Brilliant Blue Reactive and methyl red are better adsorbed at pH values lower than pH_pzc_, where the surface is positively charged [122,126]. The effect of pH is not consistent for all pollutants, as simple Coulombic expectations may be overruled by speciation. Pharmaceuticals exhibit the highest pH dependence when they exist in either a cationic or zwitterionic state and an anionic state, with amoxicillin and carbamazepine having peak removal at pH 4–5, followed by a rapid decrease in removal at alkaline pH, and tetracycline having a peak removal at pH 6–8 where it is a neutral or zwitterionic species that can interact with the π-electrons of the activated carbon [123,127]. Phenolics behave differently again: acidic pH can often increase adsorption by favoring the undissociated form (phenol), but the optimum may be towards the mildly acidic or near neutral range depending on pKa, pH_pzc_ and the role of hydrogen bonding [25,66].

### 6.2. Mass Transfer Variables

The initial pollutant concentration has a dual effect: it raises the driving force for mass transfer, and it hastens site saturation. That is why the adsorption capacity may increase as the concentration increases, while the percentage removal decreases or levels off. In *Ziziphus* systems, the removal of Cr(VI) was optimized at moderate concentrations instead of the highest concentrations tested, and in the Congo red and dye studies, concentration was used as a control variable rather than as a linear performance-enhancer [73,125]. The same trend is clear in non-*Ziziphus* analogs: methylene blue capacity rose from 14.4 to 190.2 mg g^−1^ with the increase of the concentration gradient, while the efficiency of removal of pharmaceuticals decreased at high influent concentrations due to the rapid occupation of the active sites [128]. Normally, the percentage removal will increase with increasing adsorbent dosage, as more surface area and more accessible sites are provided; however, the normalized capacity (mg g^−1^) will decrease as the same inventory of adsorbates is spread among more particles. The best Cr(VI) removal performance was obtained at 1 g/L for *Ziziphus jujuba* core AC, and 0.6 g/L for ZJS-activated biochar, indicating that the optimum dose depends on the material and is related to the texture and concentration of the target [129]. There is also a risk of particle aggregation and partial site masking as the dose is increased, which is why the performance per gram does not increase indefinitely with mass—a trend that is well documented in pharmaceutical and dye systems other than *Ziziphus* [104,130].

**Figure 9:**
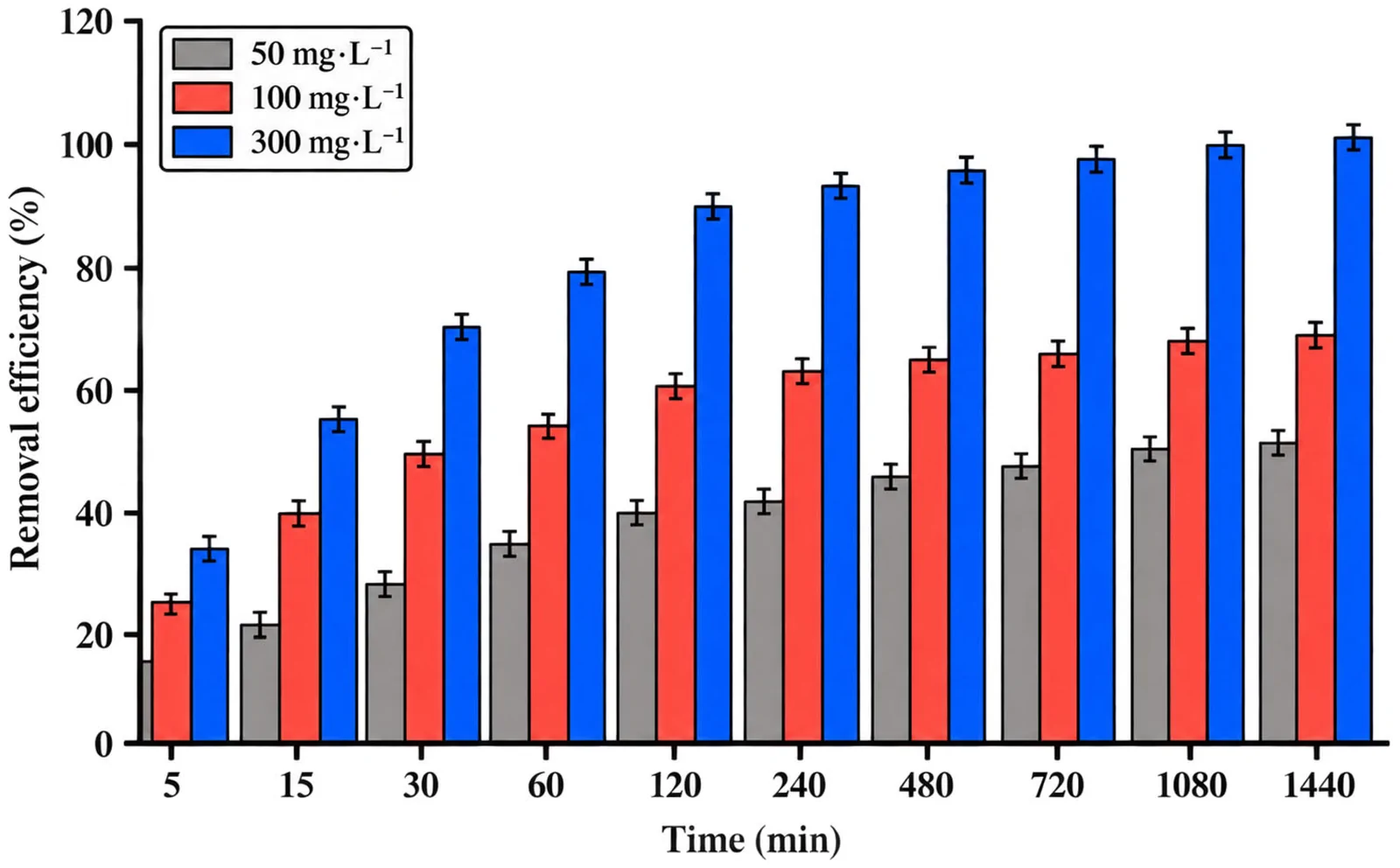
Effect of contact time on the adsorption capacity of the *Ziziphus* adsorbent across three initial adsorbate concentrations. Reproduced from Ref. [47].

The effect of contact time is more regular. Adsorption generally occurs in three steps: initial external-surface adsorption, subsequent intraparticle diffusion, and equilibrium. *Ziziphus* materials have been found to exhibit fast initial uptake and then a leveling off, such as optimum dye uptake for Indian jujube seed AC after 60 min, optimum uptake of Cr(VI) by activated ZJS biochar after 120 min, and optimum adsorption of Congo red by jujube seed carbon after 100–120 min, and intraparticle diffusion was observed but not exclusively rate-controlling [45]. This is consistent with other seed- and biochar-based carbons, where the fast stage of uptake corresponds to abundant external sites and the slow stage corresponds to the diffusion resistance and progressive site occupation [123]. As mentioned, contact time is one of the most influential parameters governing the adsorption performance of *Ziziphus* seed-based adsorbents. Figure 9 illustrates the effect of contact time on adsorption capacity at different initial adsorbate concentrations, showing rapid uptake during the initial stage followed by a slower approach to equilibrium [47].

### 6.3. Thermal and Matrix Effects

The direction of the effect of temperature on adsorption is not universal; it influences adsorption in several ways; these include adsorption energetics, viscosity and diffusivity. The majority of *Ziziphus* dye and Cr(VI) studies report endothermic behavior, meaning that increased temperature increases the mobility of the solute and accessibility to internal sites, as reported for Indian jujube seed AC, *Ziziphus jujuba* core AC and magnetic *Ziziphus*-derived systems [100,131]. However, there are exothermic systems, too. Malachite green adsorption onto sulfuric-acid-modified *Z. spina-christi* was exothermic, while the capacity of the non-seed *Ziziphus* leaves decreased with increasing temperature (25 to 45 °C) [132,133]. This discrepancy illustrates the mechanistic nature of temperature response: physisorption-dominated aromatic uptake may be mildly endothermic or exothermic depending on the hydration and pore accessibility, while metal uptake (with diffusion and activation barriers) tends to be endothermic [73,134]. Coexisting ions and ionic strength remain among the most poorly understood aspects of the *Ziziphus* seed literature. Most of the *Ziziphus*-specific studies focus on optimizing adsorption in synthetic single-solute systems and do not adequately test the effects of NaCl, hardness ions, or real wastewater competition [135,136]. Further studies indicate that adsorption of pollutants can be either inhibited or promoted depending on the type of pollutant and the mechanism of adsorption; high-salt wastewater inhibited the adsorption of methylene blue on coal-based activated carbon, with 20 g/L NaCl leading to a reduction in capacity, while for Reactive Black 5, the adsorption was enhanced by the presence of NaCl, and some cations appeared to have little effect on the adsorption on surfactant-modified activated carbon [66,137]. Competing ions are more disruptive in multicomponent systems where they compete for active sites or change speciation. Na^+^, K^+^ and Mg^2+^ inhibited tetracycline adsorption, while Ca^2+^ enhanced it by complexation; and binary pollutant systems exhibited significant antagonism due to site competition and steric effects [107,138].

### 6.4. Adsorbent Properties and Integration

The strength of the effect of operating variables is controlled by the adsorbent properties. Surface area and pore structure are significant, but not adequate predictors of performance. The BET surface area of the *Ziziphus*-derived carbons ranged from moderate (571 m^2^ g^−1^ for Indian jujube seed AC) to high (608 m^2^ g^−1^ for *Z. jujuba* core AC), but they showed significant differences in performance due to differences in pore size matching and surface chemistry. The Cr(VI) study on *Ziziphus jujuba* core AC clearly indicated that the high adsorption capacity was due to the presence of sufficient micropores suitable for adsorption of Cr(VI) species, although the BET area was only moderate. Generally, the narrower micropores control certain adsorption regimes, and the larger molecules are controlled by the mesopores for accessibility and diffusion [139,140,141]. Surface chemistry can be as important as, or more important than, the total area. The pH_pzc_, hydrogen bonding, and ion exchange capacity are regulated by oxygen-containing groups, aromatic domains, and preparation-induced acidity or basicity, thus making it possible to modify the optimum conditions and adsorption pathways by acid activation, ZnCl_2_ impregnation, and magnetic or oxide modification [142]. This also explains why raw and modified *Ziziphus* materials can show opposite thermal behavior or different equilibrium times for similar dyes. The primary implication is that these factors influence each other. The change in the charge state of the pollutant and surface is influenced by pH; the extent of this change is determined by activation chemistry. The initial concentration will affect the driving force, though the advantage will be based on the ability of the pore structure to allow diffusion without being rapidly saturated. When intraparticle diffusion is the rate-limiting step, uptake can increase with temperature, but not when the adsorption process is strongly exothermic. Laboratory optima will not necessarily be reflected in real wastewater, where salts, natural organic matter, mixed pollutants and varying pH can alter the controlling mechanism entirely [143,144]. Solution pH, adsorbent surface chemistry, and mass-transfer conditions are the most important factors that control *Ziziphus* seed adsorption, while dosage and temperature are considered system-tuning variables [143,145]. The most significant change in evidence is the transition from operating parameters as batch variables to the realization that the impacts of operating parameters are preparation dependent and pollutant specific. The most important unanswered question is how the adsorbents prepared from the *Ziziphus* seeds perform in real wastewater conditions, which are often multi-component, and this directly relates to the next section on kinetics, isotherm models and thermodynamic interpretation. Accordingly, direct comparison of adsorption capacities among *Ziziphus*-derived adsorbents should be interpreted cautiously, as variations in pH, initial concentration, adsorbent dosage, contact time, and temperature can substantially influence the reported adsorption performance.

## 7. Adsorption Models in *Ziziphus* Seed Adsorbents

The adsorption models in *Ziziphus* seed-based adsorbents answer three related questions: rate of uptake, effect of surface heterogeneity on equilibrium, and whether the adsorption is spontaneous, endothermic or exothermic. No single model is preferred for all *Ziziphus* systems in the available literature; pseudo-second-order kinetics and Freundlich isotherms are the most popular, but well-performing exceptions exist for pseudo-first-order kinetics, Langmuir monolayer behavior and mixed models like Temkin, Dubinin–Radushkevich, and Sips, based on contaminant class and adsorbent preparation [141].

### 7.1. Kinetic Models

The fitting of the kinetic models to *Ziziphus* systems often involves two distinct time regimes: a fast uptake stage and a slow diffusion-limited stage, but the fit of the model does not necessarily correspond to the mechanism. The pseudo-second-order model applies well to several *Ziziphus*-based carbons and biomass adsorbents, such as Cd^2+^ on ZnCl_2_-activated *Z. jujuba* biomass, Cr(VI) on activated *Z. jujuba* seed powder and activated biochar, Cu(II) on nano-porous shell carbon, humic acid on oxidized activated carbon and cationic dyes on H_3_PO_4_-activated Indian jujube seed carbon. In those studies, R^2^ and calculated (q_e) values were generally higher for PSO than for experiments, but this does not conclusively demonstrate chemisorption [25,142]. It is important not to overgeneralize because of important exceptions. The adsorption of indigo carmine on *Z. jujuba* biochar followed pseudo-first-order (PFO) kinetics, and Zn/Pb uptake by ultrasonic-assisted jujube-seed biochar also favored PFO, suggesting that external mass transfer or weakly bound surface uptake can dominate even in chemically modified materials [146]. The GO–*Ziziphus* dye adsorption was best described by the nonlinear pseudo-first-order model and converged to equilibrium within the first 10 min, demonstrating that hybridization can significantly reduce the apparent kinetic window [10].

Diffusion models bring in an essential caveat. In many cases, intraparticle diffusion is not the only rate-controlling step; some examples include acriflavine/Victoria blue B on IJSAC, Congo red on jujube-seed carbon and basic dyes on *Z. lotus stone* carbon [46]. Table 10 summarizes representative kinetic model fittings for *Ziziphus* seed-based adsorption systems, including the best-fitting models and key kinetic parameters, as well as their interpretation.

Equation (1) Pseudo-first-order (PFO):

$$ln(q_{e}-q_{t})={ln}_{qe}-k_{1}t$$

Equation (2) Pseudo-second-order (PSO):

$$\frac{t}{q_{t}}=\frac{1}{k_{2}{q^{2}}_{e}}+\frac{t}{q_{e}}$$

where *q_e_* and *q_t_* are adsorption capacities (mg g^−1^) at equilibrium and at time *t*, respectively; *k*_1_ is the PFO rate constant and *k*_2_ is the PSO rate constant [147].

**Table 10:** Evaluation of pseudo-first order and pseudo-second-order kinetic model fits for *Ziziphus* seed-based adsorption systems.

Adsorbent	Contaminant	Best Kinetic Model	Key Parameters	Ref.
*Z. mauritiana* seed AC	Congo red	PSO	(q_e) 9.81 mg g^−1^, (k_2) 0.182 g/mg·min, R^2^ = 1.0	[25]
Indian jujube seed AC	Acriflavine, Victoria blue B	PSO	R^2^ = 0.95–0.99	[148]
ZnCl_2_-activated *Z. jujuba* biomass	Cd^2+^	PSO	PSO governs kinetics	[32]
*Z. jujuba* core AC	Cr(VI)	PSO	R^2^ ≈ 0.995	[100]
*Z. jujuba* seed biochar	Cr (VI)	PSO	PSO best fit	[141]
Shell nano-carbon	Cu (II)	PSO	R^2^ = 0.999	[149]
Oxidized activated carbon	Humic acid	PSO	PSO best fit	[94]
*Z. jujuba* biochar	Indigo carmine	PFO	PFO best fit	[141]
Ultrasonic-assisted jujube biochar	Zn (II), Pb (II)	PFO	PFO relevant	[150]
GO–*Ziziphus* composite	Hazardous dye	PFO	Equilibrium in ~10 min	[70]

### 7.2. Isotherm Models

The equilibrium modelling reveals a clearer separation by pollutant type. Freundlich behavior is typical for metals and some dyes, suggesting heterogeneous surfaces and multilayer adsorption. The Cd^2+^ on *Z. jujuba* biomass fit Freundlich with R^2^ = 0.978, Congo red on jujube-seed activated carbon fit Freundlich with R^2^ = 0.9991 and (*n* = 1.563), humic acid on oxidized activated carbon fit Freundlich, and Cr(VI) on activated biochar fit Freundlich with R^2^ = 0.9988 and *n* = 1.559 [25,94]. The same trend was observed for Indian jujube seed carbon activated with H_3_PO_4_ for acriflavine and Victoria blue B, even though the Langmuir capacities were good (113.6 and 92.78 mg g^−1^) respectively, which shows that high capacity does not imply monolayer adsorption [45]. Langmuir fits are more common for methylene blue, Rhodamine B on raw biomass, and for some metal systems, particularly when the adsorption sites are more energetically uniform or where strong saturation behavior is observed. The activated carbon of *Ziziphus lotus* was found to fit the Langmuir equation with q max = 14.493 mg g^−1^, while the activated carbon of *Z. jujuba* seed carbon, raw Indian jujube seeds for Congo red, raw Indian jujube seeds for BY28 and raw Indian jujube seeds for BR46 fitted the Langmuir equation with q max = 142.9, 55.56, 424 and 307 mg g^−1^, respectively, with R^2^ = 0.99 [46,146]. The Langmuir and Freundlich models were also found to describe the Cu(II) adsorption on KOH-activated *Z. spina-christi* seed carbon, with a reported Langmuir capacity close to 104 mg g^−1^, showing that apparently competitive models can describe the adsorption when the range of the experiment is limited [73]. The Temkin constants suggested that the adsorption of Rhodamine B was mainly physical [144]. The Dubinin–Radushkevich model is valuable because the mean adsorption energy (E) can be used to differentiate between physical adsorption (E < 8 kJ/mol) and ion-exchange-like behavior; however, the model is used qualitatively in *Ziziphus* studies rather than in strong comparisons [100]. Sips and Redlich–Peterson sometimes outperformed classical two-parameter models for Cr(VI); the activated *Z. jujuba* seed powder gave Freundlich R^2^ = 0.9926, and Sips and Redlich–Peterson fits were close to 0.97–0.995, whereas a different Cr(VI) system favored Tóth and Elovich, indicating that flexible empirical models can capture the heterogeneity better than Langmuir/Freundlich alone [48]. To better understand the adsorption process, numerous studies have applied equilibrium isotherm models to *Ziziphus* seed-derived adsorbents, as summarized in Table 11. Comparison of the reported equilibrium models reveals that model preference is dependent on both adsorbate class and adsorbent surface characteristics. Freundlich behavior is frequently reported for *Ziziphus*-derived heterogeneous carbons, particularly where chemically modified surfaces contain energetically non-uniform binding sites. In contrast, Langmuir behavior is observed in systems showing stronger saturation characteristics, where adsorption can be represented by a finite population of relatively uniform sites. For heavy-metal ions, the occurrence of Freundlich behavior is consistent with the coexistence of several possible interactions, including complexation, ion exchange and electrostatic attraction, whereas Langmuir fits indicate that a more restricted population of dominant binding sites may control uptake [151,152]. Dye adsorption similarly varies with molecular structure, surface charge and pore accessibility; consequently, both Langmuir and Freundlich behavior have been reported across *Ziziphus*-derived adsorbents. Therefore, the available evidence does not support assigning a single equilibrium model to all dyes or all metals. Instead, model preference reflects the combined influence of adsorbate chemistry, surface heterogeneity and adsorbent preparation.

Equation (3) Freundlich:

$$q_{e}=K_{F}\;C_{e}^{\left(\frac{1}{n}\right)}\;$$

where $q_{e}$ is the equilibrium quantity of adsorbate on the adsorbent (mg g^−1^). The Freundlich constant, *K_F_*, represents adsorption capacity and has units of (mg g^−1^) (L mg^−1^)^1/*n*^, while 1/*n* is a dimensionless parameter describing adsorption intensity and surface heterogeneity. A value of 1/*n* between 0 and 1 is commonly interpreted as indicating favorable adsorption, whereas 1/*n* > 1 may indicate less favorable adsorption under the conditions investigated.

Equation (4) Langmuir:

$$\frac{C_{e}}{q_{e}}=\frac{1}{K_{L}.Q_{max}}+\frac{C_{e}}{Q_{max}}$$

where $q_{e}$ is the equilibrium amount of adsorbate in the adsorbent (mg g^−1^). $Q_{max}$ is the maximum capacity for monolayer coverage (mg g^−1^). $K_{L}$ is the constant of the Langmuir isotherm (L/mg). $C_{e}$ is the equilibrium concentration of adsorbate (mg L^−1^).

### 7.3. Thermodynamics

Thermodynamic analyses agree on one thing with the isotherm literature: adsorption on the *Ziziphus*-derived materials is generally spontaneous, as indicated by the negative values of ΔG° for a variety of dyes, metals, and types of natural organic matter [155,156]. However, the sign of ΔH° varies. Endothermic behavior is observed with indigo carmine on *Z. jujuba* biochar, acriflavine and Victoria blue B on IJSAC, humic acid on oxidized activated carbon, Cr(VI) on *Z. jujuba* core AC, methylene blue on *Z. lotus* core AC, Cu(II) on shell nano-carbon, and Rhodamine B on ZnCl_2_-activated carbon [157]. For these systems, a positive ΔH° typically means that the adsorption increases with temperature, which is frequently a result of the diffusion of molecules into the pores or activation of surface sites becoming more favorable with increasing temperature. Exothermic systems are not uncommon, however. The removal of Zn(II) and Pb(II) by ultrasonic-assisted jujube-seed biochar was feasible, spontaneous, and exothermic, and so was the dye adsorption by GO–*Ziziphus*, while the adsorption of Pb(II) by chemically modified *Ziziphus* biomass was spontaneous and exothermic, with increasingly negative ΔG° as the temperature increased [132]. These differences warn against assuming that ΔH° is a material property; it will vary depending on the hydration of the pollutant, the binding mode, and the relative roles of diffusion and destabilization of weak surface associations at the interface. The ΔS° is less consistently reported, but when it is, it typically shows a gain in interfacial disorder during adsorption. Although the adsorption of Rhodamine B was exothermic, a positive ΔS° indicated increased randomness at the solid–liquid interface, and the adsorption of Pb(II) on chemically modified *Ziziphus* biomass was also exothermic, but with positive ΔS° [132,154]. Thermodynamic interpretation therefore needs to be read alongside kinetics and isotherms rather than in isolation.

The reported thermodynamic behavior varies considerably among the *Ziziphus*-derived adsorbents in Table 12. The Cr(VI) adsorption on Rb_2_CO_3_-activated carbon and cationic dye adsorption on H_3_PO_4_- and H_2_SO_4_-activated carbons were predominantly endothermic and entropy-driven, whereas PTZS-CN adsorption on the GO–*Ziziphus* composite was exothermic. The negative ΔG° values observed for most systems indicate favorable adsorption under the investigated conditions, although Cr(VI) adsorption became spontaneous only above approximately 295 K. These differences demonstrate that thermodynamic behavior depends on the adsorbate, surface modification, and experimental conditions rather than on the *Ziziphus* precursor alone.

In general, the adsorption models indicate that the adsorption properties of *Ziziphus* seed-based adsorbents do not form a single family with a single kinetic and equilibrium signature. PSO kinetics and Freundlich isotherms are most common, particularly for heterogeneous carbons and metal adsorption, while PFO, Langmuir, and flexible three-parameter models are important for rapidly adsorbing dyes, strongly saturating systems, or structurally hybrid adsorbents. Finally, thermodynamic data complete the picture, revealing that spontaneous adsorption may be endothermic or exothermic, depending on the preparation history that influences the pore accessibility and surface binding. This model-based variability sets up the next section, which compares kinetic fits, equilibrium capacities and thermodynamic parameters as predictors of practical wastewater performance and process design.

## 8. Adsorption Performance

The most important evidence for heavy metals, particularly Cr(VI), is that preparation clearly regulates uptake and deployability. Magnetic *Ziziphus jujuba* seed biochar also achieved high removal efficiency of 98% at 60 mg L^−1^, pH 2, 0.6 g L^−1^, 30 °C and 120 min, with a regeneration efficiency of >95%, by using 0.3 M HCl. The magnetic route is more convincing than the batch data alone, as magnetic biochar achieved 99% Cr(VI) removal at 6 cm bed height, 60 ppm influent, and 20 mL min^−1^ [158,159]. Compared with the chromium studies, the literature on other metals provides more information on selectivity between metals. The raw jujube seeds were treated with sulfuric acid and ultrasound, and the resulting Langmuir capacities for Zn(II) and Pb(II) were 221.1 mg g^−1^ and 119.8 mg g^−1^, respectively, which were higher than those of the raw precursor; the material could be reused for five adsorption–desorption cycles [146]. In contrast, phosphoric-acid-derived stem carbons had a higher adsorption capacity for Pb(II) than Cd(II), and the unoxidized sample had a higher capacity than the oxidized sample, indicating that introducing oxygen-containing functional groups does not necessarily increase the metal adsorption capacity [160]. In general, the adsorption of heavy metals on biochar is influenced by complexation, ion exchange, cation-π bonding, electrostatic effects, and precipitation, and, therefore, the capacity of *Ziziphus* materials should not be interpreted based on surface area only [161]. There is another hierarchy for organic dyes. Conventional acid-activated carbons are capable but rarely outstanding: Indian jujube seed carbon activated with H_3_PO_4_ showed good adsorption capacity for acriflavine (113.6 mg g^−1^) and Victoria blue B (92.78 mg g^−1^), with optimum adsorption under alkaline conditions and a relatively short equilibration time [46]. Another study correlated the increased porosity and acidity of ortho-phosphoric-acid-modified *Ziziphus jujuba* stones with better performance for methylene blue (179.83 mg g^−1^) [127]. Raw Indian *jujube* seeds still removed Congo red up to 55.56 mg g^−1^, but only under strongly acidic conditions, which makes them more attractive as a low-cost proof of concept than as a robust process adsorbent [162,163]. The highest dye capacities come from hybrid materials, not from seed carbon alone. OA-JS@ZnFe_2_O_4_ nanocomposite achieved 980.39 mg g^−1^ for Congo red and 476.18 mg g^−1^ for methylene blue and still retained 82.08% and 76.59% removal after seven cycles [164]. GO–*Ziziphus* followed the opposite pattern: its reported dye capacity was only 34 mg g^−1^, but adsorption was rapid, best fit by nonlinear pseudo-first-order kinetics, and thermodynamically spontaneous and exothermic [159]. That contrast is important. Some modifications maximize capacity, whereas others improve kinetics, regeneration, or handling, without producing record equilibrium uptake.

### 8.1. Nutrients and Emerging Contaminants

The most obvious gap in the *Ziziphus* seed literature is nutrient removal. No *Ziziphus* papers report NH_4_^+^, NO_3_^−^, or PO_4_^3−^ adsorption, and a strong nutrient-performance section would be difficult to write from data from just the seeds. The biochar literature in general suggests that unmodified biochar typically exhibits NH4-N capacities of less than 20 mg g^−1^ and even lower affinity for NO_3_^−^ due to its negative charge that favors repulsion with the biochar, and that only metal- or metal-oxide-modified biochars have significant phosphate capture ability [157]. This is important here since most of the high-performance *Ziziphus* adsorbents have been optimized for dyes or metals, rather than deliberately designed for nutrient capture. The emerging contaminant story is better but not uniform. *Ziziphus*-derived biochar has already shown a very high ability to remove nylon and polyethylene microplastics, with both BC300 and BC700 achieving over 99% removal, and BC700 achieving 33 times the removal of sand [157]. In the same study, biochar loaded with microplastic was then used as a second-stage adsorbent, in which the biochar treated with BC700/PE was used for the removal of Pb(II) and Cd(II), with a capacity of 584.34 mg g−1 and 552.80 mg g−1, respectively. It is one of the most interesting results in the corpus, as it goes beyond testing with a single solute and uses a *Ziziphus*-derived material in integrated co-contaminant treatment.

The evidence is still circumstantial for pharmaceuticals and PFAS. Both reviews agree that biochar with little or no processing has a limited spectrum of adsorbing contaminants and typically requires some form of processing to be competitive with other materials [157,165]. An effective analogue of the seed outside *Ziziphus* is the biochar derived from litchi seeds modified with ZnCl_2_, which achieved removal efficiencies >95% for all tested PFASs, was stable in the pH range 3–9, and maintained its activity after five rejuvenation cycles in real waters [85].

### 8.2. Critical Comparison

Collectively, adsorption performance is influenced more strongly by adsorbent modification and physicochemical properties than by pollutant class alone. Promising batch, regeneration, and limited column-scale results have been reported for heavy-metal removal, particularly for Cr(VI) and Zn/Pb systems [96]. For example, magnetic *Z. jujuba* biochar achieved 98–99% Cr(VI) removal [158,159], while H_2_SO_4_/ultrasonic-treated seeds reached 221.1 and 119.8 mg g^−1^ for Zn(II) and Pb(II), respectively [146]. For dyes, H_3_PO_4_-activated seed carbon achieved 113.6 and 92.78 mg g^−1^ for acriflavine and Victoria blue B, respectively [46]. These differences highlight the influence of adsorbent preparation and modification, although direct comparison remains limited by variations in experimental conditions. Nevertheless, most studies have been conducted using laboratory-scale model solutions, limiting the assessment of performance under realistic treatment conditions [166]. The current evidence is concentrated primarily on metals and dyes, while nutrients, pharmaceuticals, PFAS, and other emerging contaminants remain largely underexplored [158]. Additional challenges include uncertain long-term regeneration and adsorbent stability, chemical and energy demands associated with activation and modification, potential leaching of modifying agents, and secondary waste generation during regeneration. Further studies using real wastewater, continuous-flow systems, and scale-up evaluation are therefore required to establish the practical applicability of *Ziziphus* seed-derived adsorbents.

### 8.3. Regeneration

Regeneration and reusability are essential for evaluating the practical sustainability and circular-economy potential of *Ziziphus*-derived adsorbents. Reported performance varies among materials: ZnCl_2_-activated *Z. jujuba* seed biomass was reusable for four cycles [32], while H_3_PO_4_-activated *Z. spina-christi* carbon was evaluated over five regeneration cycles [46]. Magnetic *Z. jujuba* seed biochar achieved >95% Cr(VI) desorption using 0.3 M HCl [54], whereas the OA-JS@ZnFe_2_O_4_ nanocomposite retained 82.08% and 76.59% removal of Congo red and methylene blue, respectively, after seven cycles [164]. Although these results demonstrate promising reuse potential, regeneration can generate concentrated secondary waste and may cause adsorbent deterioration or leaching of residual modifying agents. Moreover, chemical activation and composite modification can increase chemical and energy consumption, washing requirements, and secondary waste generation. Therefore, future studies should integrate regeneration, leaching, waste management, and techno-economic and life-cycle assessments to establish the practical sustainability and circularity of these materials.

## 9. Conclusions and Future Perspectives

This critical review evaluates the material preparation, surface functionalization, adsorption mechanisms, and water-treatment applications of *Ziziphus* seed-based adsorbents. Mechanistic and thermodynamic analyses show that *Ziziphus* seeds provide a highly viable, structurally robust lignocellulosic platform but cannot be considered a universal high-performing adsorbent. The most fundamental finding from the recently published literature is that the adsorption efficacy is determined primarily by the strategic thermochemical and chemical transformation of the feedstock, rather than by botanical origin alone; the former includes mineral acid activation, alkali activation, magnetization, and nanocomposite hybridization. The carbons engineered in this way are systematically modified with respect to specific surface area, hierarchical pore distribution, surface charge and the presence of oxygenated functional groups and are thus very effective for sequestering traditional inorganic and organic pollutants such as heavy metals (Cr(VI), Cd(II), Pb(II)) and synthetic dyes. Among the reported approaches, H_3_PO_4_ activation resulted in highly porous *Ziziphus*-derived carbon, reaching a surface area of 846 m^2^ g^−1^ at a 2:1 impregnation ratio and 500 °C, with effective adsorption of cationic dyes. Alkali and magnetic modifications also enhanced the removal of heavy metals, including Cr(VI), Zn(II), and Pb(II). Nevertheless, differences in preparation and experimental conditions, together with limited real-water and continuous-flow studies, restrict direct comparison and assessment of practical performance. In addition, there are regional and economic benefits that can be derived from the valorization of species that are widely distributed, such as *Z. mauritiana* and *Z. spina-christi*, in the arid and semi-arid regions. The use of these agricultural residues is directly related to the principles of the circular bioeconomy, and comprise a strategy aimed at repurposing local solid waste that would otherwise be discarded, creating low-cost sources of decentralized water security. Although much good work has been accomplished at the bench scale, the overall situation of adsorption research on *Ziziphus* is not yet very mature and is still plagued by many methodological limitations. The main limitation is the excessive use of idealized configurations (batch, single solute) with highly optimized operating conditions. This restricted approach cannot address the competitive adsorption, ionic screening, steric hindrance, and pore-blocking phenomena that can occur when multiple ions are present in a real, multicomponent wastewater. Moreover, the targeted contaminant envelope is still disproportionately skewed towards legacy contaminants. Empirical data on recovery of nutrients such as phosphate and ammonium, and elimination of emerging contaminants such as recalcitrant pharmaceuticals, microplastics, personal care products and per- and polyfluoroalkyl substances (PFAS), are lacking. Although preliminary studies on microplastic entrapment and co-adsorption of heavy metals on biochar are promising, there is not a well-established mechanism for highly polar or persistent micro-pollutants in the *Ziziphus* literature.

Future research needs to shift to a mechanism-based, scale-aware design paradigm to advance *Ziziphus*-based adsorbents to field-deployable environmental technologies. Three important areas should be investigated in the future. First, the surface functionalization should be a highly targeted process, and the researchers should balance the incremental benefits of surface adsorption capability with the cost of synthesis, the toxicity of the reagents, and the burden of wash water to the environment that can be caused by complex functionalization procedures. Second, a shift to continuous flow systems is a necessity. Realistic multicomponent effluents will yield a much more realistic measure of practical viability than equilibrium batch tests when evaluating breakthrough behavior, mass-transfer zones, and hydrodynamic stability in fixed-bed columns. Third, the adsorption–desorption cycle needs to be tested over long periods to establish mechanical stability, reduce risks of the secondary pollution caused by the leaching out of active-phase components (e.g., magnetic iron oxides or grafted transition metals), and define sustainable end-of-life management strategies for exhausted sorbents.

## Figures and Tables

**Table 1:** Key physical descriptors of *Ziziphus* seed-derived activated carbons and their relevance to adsorption performance.

Precursor and Product	Key Physical Descriptor	Adsorption-Relevant Implication	Ref.
*Z. mauritiana* seed AC (H_3_PO_4_)	571 m^2^/g, mesoporous, pore radius 22.45 Å	Strong dye uptake, heterogeneous surface	[46]
*Z. spina-christi* ACJS2 (H_3_PO_4_)	846 m^2^/g, 0.828 cm^3^/g, pH_pzc_ 4.03	High mesoporosity and acidic surface	[47]
*Z. lotus* core AC (H_2_SO_4_)	749.6 m^2^/g, pores 10–45 μm	Fast MB uptake, carboxylic/acidic surface	[47]
*Z. lotus* AC for Cd/Cr(VI)	Pores 6–50 μm	Rapid metal uptake in 80 min	[48]
Fe_3_O_4_/SDS-decorated *Ziziphus* AC	102.04 m^2^/g, 169.49 Å pores	Lower area, but boosted Cr^3+^ capture via functionalization	[49]

**Table 2:** Chemical characterization of distinct *Ziziphus* seed precursors and their corresponding functional utility in adsorption.

Species	Reported Seed Chemistry	Likely Adsorbent Relevance	Ref.
*Z. mauritiana*	Crude fiber 48.12%, carbohydrate 63.24%, fat 1.89%	Strong lignocellulosic backbone, low lipid burden	[43]
*Z. jujuba var. spinosa*	114 compounds in seeds, rich in flavonoid glycosides and saponins	Chemically diverse precursor, likely rich in oxygenated moieties before pyrolysis	[39]
*Z. spina-christi stones*	Protein, crude fat, fibers, carbohydrates, sugars, macroelements	Mixed organic–mineral matrix for complexation and ion exchange	[29]

**Table 3:** Comparative evaluation of *Ziziphus* seeds and major agricultural waste precursors for scalable adsorbent engineering.

Biomass	Main Advantage	Main Limitation	Comparative Note	Ref.
*Z. lotus* nucleus-derived activated carbon	Effective removal of Cd(II) and Cr(VI) using low-cost biomass precursor	Limited validation with real wastewater and relatively narrow contaminant range	Achieved about 60% Cd(II) and 80% Cr(VI) removal after 80 min, outperforming coffee-ground activated carbon	[48]
Date seed (Phoenix dactylifera)	Low-cost biomass precursor; Khalas AC reached 165 mg g^−1^ MB capacity	Strong dependence on seed cultivar and activation conditions	Best-performing Khalas AC achieved 165 mg g^−1^, equivalent to 71% of CAC capacity	[59]
Coconut shell	High BET area, 1000.15 m^2^ g^−1^; high fixed carbon, 88.92%; 96% MB removal	Often needs stronger activation	Best overall performer among five agricultural wastes; MB capacity 80 mg g^−1^	[58]
Rice husk	Low-cost, renewable and applicable to several pollutant classes	Adsorption performance is strongly treatment-dependent; simple chemical treatment gave only 3.2 m^2^ g^−1^ surface area	Thermal treatment increased BET area to 24–201 m^2^ g^−1^ and achieved 36–64% phenol removal, compared with 28% after chemical treatment	[62]
Peanut shell	High dual dye/metal adsorption, with >95% removal	Depends on activation method and activator ratio	303.03 mg g^−1^MB and 130.89 mg g^−1^Pb(II)	[63]

**Table 4:** Summary of representative *Ziziphus* seed-based adsorption studies (2020–2025) and their key mechanistic findings.

Year	*Ziziphus* Material	Pollutant	Capacity/Performance	Key Finding	Ref.
2020	Modified *Z. spina-christi* stones	Pb(II), Zn(II), Cd(II)	9.06, 4.19, 5.38 mg g^−1^	KOH modification outperformed acidic treatments	[29]
2021	ZnCl_2_-activated *Z. jujuba* seed biomass	Cd^2+^	49.40 mg g^−1^	Freundlich + pseudo-second order; reusable for 4 cycles	[32]
2022	H_3_PO_4_-activated *Z. mauritiana* seed AC	Acriflavine, Victoria blue	113.6, 92.78 mg g^−1^	Mesoporous AC; dye uptake via H-bonding, electrostatic, π–π	[46]
2022	H_2_SO_4_-activated *Z. lotus* core AC	Methylene blue	14.493 mg g^−1^	Fast uptake, porous acidic surface	[47]
2023	GO–*Ziziphus* seed composite	PTZS-CN dye	29.03 mg g^−1^	Freundlich, pseudo-first-order, physical and exothermic adsorption	[10]
2023	H_3_PO_4_-activated *Z. spina-christi* ACJS2	Tartrazine	160 mg g^−1^	Mesoporous acidic AC; five-cycle regeneration	[47]
2024	Zn/ZMSAC composite	Rhodamine B, Orange II	Efficient removal reported	Synergistic adsorption–photocatalysis	[54]
2024	*Z. lotus* AC	Cd and Cr (VI)	60% Cd, 80% Cr (VI) removal	More effective than coffee-ground AC	[48]
2025	Magnetic biochar from *Z. jujuba* seed	Cr (VI)	98% removal	Langmuir, pseudo-second-order, >95% desorption recovery	[61]

**Table 5:** Chemical activation routes for *Ziziphus* seed-derived materials and their functional outcomes in wastewater treatment applications.

Activator	*Ziziphus* Precursor	Preparation Highlights	Key Physicochemical Outcome	Adsorption Outcome	Ref.
H_3_PO_4_	*Z. mauritiana seeds*	Chemical activation to AC	571 m^2^/g, mesoporous	113.6 mg g^−1^AF, 92.78 mg/g VB	[46]
H_3_PO_4_	*Z. spina-christi seeds*	2:1 impregnation, 500 °C	846 m^2^/g, 0.828 cm^3^/g, pH_pzc_ 4.03	160 mg g^−1^ tartrazine	[47]
H_3_PO_4_	*Z. jujuba* stones	Chemical activation	1896 m^2^/g, acidic surface	37.04 mg g^−1^BRSM dye	[65]
H_2_SO_4_	*Z. lotus* cores	24 h activation, 500 °C, 2 h	749.6 m^2^/g, acidic/carboxylic surface	14.493 mg g^−1^MB	[48]
H_2_SO_4_	*Z. spina-christi* shell seeds	3:1 acid mixing, 150–200 °C, 4 h	Modified pore structure and functionality	MG from 48.78 to 370.37 mg g^−1^	[66]
ZnCl_2_	*Z. jujuba* waste seeds	Chemical activation biomass sorbent	Heterogeneous surface for Cd^2+^ uptake	49.40 mg g^−1^Cd^2+^; 4-cycle reuse	[32]
KOH/citric/HCl/H_2_O_2_	*Z. spina-christi* stones	Surface modification rather than full AC	Binding-site exposure varies by reagent	KOH best overall for Pb, Zn, Cd	[29]

**Table 6:** Functional advantages and inherent limitations of structurally modified *Ziziphus* nanocomposites and magnetic biochar.

Modified Material	Modifier/Strategy	Main Property Gained	Pollutant Target	Performance	Limitation	Ref.
GO–*Ziziphus*	Graphene oxide composite	Large surface area, 2209 m^2^ g^−1^; heterogeneous, oxygen-functionalized surface	PTZS-CN dye	29.03 mg g^−1^	Multi-step, chemical- and energy-intensive synthesis	[10]
Magnetic biochar	Magnetic phase incorporation	Easy recovery, FeO-containing surface	Cr (VI)	98% batch, 99% column	More complex synthesis	[54]
Zn/ZMSAC	ZnO loading by hydrothermal route	Adsorption–photocatalysis synergy	Rhodamine B, Orange II	Efficient dye removal	Added metal cost and process complexity	[54]
KOH-modified stones	Alkali surface treatment	More carboxylate binding sites	Pb (II), Zn (II), Cd (II)	Best among tested modifiers	Lower textural control than AC routes	[29]

**Table 7:** Principal physicochemical characterization techniques and their diagnostic utility in *Ziziphus* seed adsorption studies.

Technique	Purpose	Information Obtained
SEM	Visualize morphology and pore development	Surface roughness, cracks, pore opening, pore filling after adsorption; *Ziziphus* carbons showed porous surfaces with pores from 6–50 μm in reported studies [48]
EDS/EDX	Confirm elemental composition and adsorbate loading	Presence of elements before and after adsorption, including confirmation of Cr on *Ziziphus* seed adsorbent surfaces [48]
BET/N_2_ Sorption	Quantify textural properties	Specific surface area, pore volume, pore-size distribution; reported *Ziziphus*-derived carbons reached 571–970 m^2^/g in the cited seed-related studies [91]
FTIR	Identify functional groups involved in adsorption	Carboxyl, acidic, oxygen-containing groups; supports roles for complexation, H-bonding, electrostatic attraction, and π–π interactions [47]
XRD	Assess crystallinity and mineral phases	Broad peaks indicate predominantly amorphous carbon; can also detect ash/mineral residues [48]
XPS	Determine surface elemental states and redox chemistry	Oxidation states and bonding environments; useful for distinguishing Cr(VI) and Cr(III) and establishing redox-assisted adsorption mechanisms [96]
Zeta Potential/pH_pzc_	Evaluate pH-dependent surface charge	Electrostatic attraction/repulsion behavior; acidified surfaces can show higher zeta potential and stronger anion uptake, while ACJS2 had pH_pzc_ 4.03 [47]

**Table 8:** Dominant adsorption pathways, corresponding controlling variables, and application scope in *Ziziphus* seed-based environmental remediation.

Mechanism	Main Controlling Variables	Strongest Relevance for *Ziziphus* Systems	Ref.
Electrostatic attraction	pH, pH_pzc_, zeta potential, pollutant charge	Cationic dyes at neutral-alkaline pH; Cr(VI) at acidic pH	[100]
Ion exchange	Exchangeable cations, acidic groups, ionic strength	Heavy metals and oxyanions	[29]
Surface complexation	–OH, –COOH, N/P/S groups, metal hardness/softness	Pb, Cd, Zn species	[29]
π–π interaction	Aromaticity, graphitic domains, electron donor/acceptor balance	Aromatic dyes, phenolics, pharmaceuticals	[101]
Pore filling	BET area, micro/mesoporosity, adsorbate size	Dyes and aromatic organics after activation	[102]

**Table 9:** Effect of key operating conditions on the adsorption capacities of *Ziziphus* seed-based systems.

*Ziziphus* Adsorbent	Pollutant	pH Effect	Contact Time	Temperature	Dosage/Concentration/Ionic Strength	Ref.
*Z.mauritiana* seed AC (H_3_PO_4_)	Acriflavine, Victoria blue B	Optimum at pH 8–10	60 min optimum	Endothermic	Dose 1.5–2.5 g/L; concentration 130–140 mg L^−1^	[46]
*Z. jujuba* seed activated biochar	Cr(VI)	Optimum at pH 2	120 min	Best at 30 °C in study	0.6 g/L; 60 mg L^−1^ initial Cr	[76]
Magnetic *Z. jujuba* seed biochar	Cr(VI)	pH, dosage, concentration, time all controlled uptake	120 min	Not detailed in excerpt	Batch controlled by four variables	[68]
*Z. jujuba* core AC	Cr(VI)	Optimum at pH 2	pH-dependent kinetics	Endothermic, 20–40 °C	Best at 1 g L^−1^ and 100 mg L^−1^	[100]
*Z. spina-christi* shell seed AC	Malachite green	pH-sensitive dye removal	Equilibrium assessed up to 6 h; kinetics followed pseudo-second-order	Raw system endothermic; acid-modified exothermic	Adsorbent dose and initial MG concentration significantly influenced uptake; isotherm tests included about 35 mg L^−1^ for SZC and 200 mg L^−1^ for SZC-AC	[25]
*Z. mauritiana* seed AC	Congo red	pH fixed at 7.2; pH effect not investigated	~120 min AC equilibrium	30 °C tested	Initial concentration and dose studied	[125]

**Table 11:** Equilibrium adsorption isotherm parameters and best-fit models for *Ziziphus* seed-derived materials.

Adsorbent	Contaminant	Langmuir Parameters	Freundlich Parameters	Best Fit	Ref
*Z. jujuba* biochar	Indigo carmine	166.46 mg g^−1^ K_L_ = 0.094	R^2^ = 0.974 1/*n =* 0.660	Freundlich	[32]
ZnCl_2_-activated *Z. jujuba* biomass	Cd^2+^	49.40 mg g^−1^ K_L_ = 0.003	R^2^ = 0.978 1/*n* = 0.222	Freundlich	[32]
Indian jujube seed AC	Acriflavine/Victoria blue B	113.6 mg g^−1^ K_L_ = 0.010	R^2^ = 0.99 1/*n* = 0.24	Freundlich	[153]
*Z. mauritiana* seed AC	Congo red	Not primary best-fit metric reported	K_F_ 19.73, *n* 1.563, R^2^ = 0.9991	Freundlich	[25]
Oxidized activated carbon	Humic acid	76.92 mg g^−1^ K_L_ = 0.004	*n =* 1.59	Freundlich	[94]
*Z. jujuba* core AC	Cr(VI)	Not best overall; Tóth best	Not primary best-fit metric	Both	[100]
Activated *Z. jujuba* seed powder	Cr(VI)	9.402 mg g^−1^ K_L_ = 0.343 R^2^ = 0.9704	K_F_ 2.541, *n* 2.963, R^2^ = 0.9926	Freundlich/R-P	[48]
*Z. lotus* core AC	Methylene blue	14.493 mg g^−1^, R^2^ = 0.982 K_L_ = 1.50	K_F_ 6.46, 1/*n* = 0.926 R^2^ = 0.899	Langmuir	[86]
ZnCl_2_-activated *Z. jujuba* seed AC	Rhodamine B	142.9 mg g^−1^ K_L_ = 0.19 R^2^ = 0.924	1/*n* =0.29	Langmuir	[154]
*Z. lotus* stone AC	BY28/BR46	424/307 mg g^−1^, R^2^ = 0.99	Not best	Langmuir	[154]
Raw Indian jujube seeds	Congo red	55.56 mg g^−1^ K_L_ = 3.1847 R^2^ = 0.999	Fitted, not best K_F_ = 4.69	Langmuir	[33]

**Table 12:** Thermodynamic parameters reported for *Ziziphus*-derived adsorbents. ^a^ Value originally reported in J mol^−1^; ^c^ ΔH° and ΔS° vary with initial dye concentration; ^d^ ΔS° was reported with an apparent unit error and is presented here as J mol^−1^ K^−1^.

Adsorbent	Adsorbate	T Range (K); Equilibrium Constant Used	ΔG° (kJ mol^−1^) at 303, 313, 323 K	ΔH° (kJ mol^−1^) ΔS° (J mol^−1^ K^−1^)	Nature of the Process
Rb_2_CO_3_-activated *Z. jujuba* core AC	Cr(VI) (oxyanion)	293–313; K_c_ = (C_0_ − C_e_)/C_e_	+0.42 (293 K) −1.15 (303 K) −2.74 (313 K)	+46.8 +158.5	Endothermic, entropy-driven; non-spontaneous at 293 K (crossover ≈ 295 K) [100]
GO–*Ziziphus* seed composite	PTZS-CN (cationic dye)	298–328; K_d_ = q_e_/C_e_	−38.7 (298 K) −37.7 (308 K) −36.2 (318 K) −34.1 (328 K)	−84.3 ^a^ −152.3	Exothermic, enthalpy-driven; inconsistent with the D–R energy of 1.02 kJ mol^−1^ reported in the same study [10]
H_3_PO_4_-activated *Z. mauritiana* seed AC (IJSAC)	Acriflavine (cationic dye)	303–323;	−4.67, −5.10, −5.57	+7.88 +41.5	Endothermic, entropy-driven, spontaneous throughout (crossover 190–221 K); internally consistent, but ΔH° and ΔS° fall as C_0_ rises ^c ^ [46]
H_3_PO_4_-activated *Z. mauritiana* seed AC (IJSAC)	Victoria blue B (cationic dye)	303–323;	−7.44, −7.85, −8.37	+6.94 (150) +47.4 (150)	Endothermic, entropy-driven, spontaneous throughout (crossover 147–193 K); stronger affinity than AF (more negative ΔG°) despite a lower Langmuir capacity. [46]
H_2_SO_4_-activated *Z. lotus* core AC (CNZL)	Methylene blue (cationic dye)	293–323; K_d_ = q_e_/C_e_	−1.93 (293 K), −6.23 (323 K)	+40.1 +143.4 ^d^	Endothermic, entropy-driven, spontaneous throughout (crossover ≈ 280 K) [48]

## Data Availability

No new data were created or analyzed in this study. Data sharing is not applicable to this article.

## Abbreviations

The following abbreviations have been used in this manuscript:

AF	Acid Fuchsin
VB	Victoria Blue
MB	Methylene Blue
TZ	Tartrazine
PTZS-CN	2-((10-Octyl-9,10-dihydroanthracen-2-yl)methylene)malononitrile
ACJS2	Activated Carbon Z. seeds
